# Risk Assessment Using Risk Scores in Patients with Acute Coronary Syndrome

**DOI:** 10.3390/jcm9093039

**Published:** 2020-09-21

**Authors:** Dean Chan Pin Yin, Jaouad Azzahhafi, Stefan James

**Affiliations:** 1 Department of Cardiology, St. Antonius Hospital Nieuwegein, 3435CM Nieuwegein, The Netherlands; j.azzahhafi@antoniusziekenhuis.nl; 2 Department of Medical Sciences and Uppsala Clinical Research Center, Uppsala University Hospital, 751 85 Uppsala, Sweden; stefan.james@ucr.uu.se

**Keywords:** acute coronary syndrome, myocardial infarction, risk scores, treatment decision-making

## Abstract

Risk scores are widely used in patients with acute coronary syndrome (ACS) prior to treatment decision-making at different points in time. At initial hospital presentation, risk scores are used to assess the risk for developing major adverse cardiac events (MACE) and can guide clinicians in either discharging the patients at low risk or swiftly admitting and treating the patients at high risk for MACE. During hospital admission, risk assessment is performed to estimate mortality, residual ischemic and bleeding risk to guide further in-hospital management (e.g., timing of coronary angiography) and post-discharge management (e.g., duration of dual antiplatelet therapy). In the months and years following ACS, long term risk can also be assessed to evaluate current treatment strategies (e.g., intensify or reduce pharmaceutical treatment options). As multiple risk scores have been developed over the last decades, this review summarizes the most relevant risk scores used in ACS patients.

## 1. Introduction

Acute coronary syndrome (ACS), including unstable angina (UA), non-ST-segment elevation myocardial infarction (NSTEMI) and ST-segment elevation myocardial infarction (STEMI), is one of the most frequent reasons for cardiac hospital admission [1]. For every ACS patient, a risk assessment is performed prior to treatment decision-making at different points along the ACS pathway [2,3,4,5]. Based on clinical parameters, multiple risk scores have been developed to aid physicians in risk stratification in complement to clinical judgement. Though no score is perfect, risk scores are still considered as valuable tools in clinical decision-making.

Since the widely used Framingham risk score was published as a prediction model for incident cardiovascular disease, numerous risk prediction models used in cardiovascular medicine have been developed over the last few decades [6]. Risk models are often developed from predictors identified from large datasets and afterwards are simplified into risk scores, where numerical weights (points) are assigned to each risk factor, which reflects the risk of a certain outcome. This allows the risk scores to be easily calculated and interpreted, and some risk scores are also used as decision rules to aid physicians in daily clinical practice.

At different moments in the timeline of ACS, risk scores can be used for risk stratification. At initial presentation, risk scores are used in patients with acute chest pain and possible ACS to, first, identify patients at low risk for major adverse cardiac events (MACE) and second, as a decision rule to swiftly discharge these patients without additional invasive testing. At admission, risk scores are used to estimate the in-hospital or short-term risk of mortality and/or bleeding and to guide further in-hospital management (e.g., timing of coronary angiography). Individual risk of mortality, myocardial infarction (MI) or bleeding may be re-evaluated before hospital discharge to determine the outpatient treatment strategy. In the months or years following ACS, risk scores are available to calculate ischemic-bleeding trade-off risk for optimal antithrombotic management or to estimate long term risk for future cardiovascular events. Using this ACS timeline, the most relevant risk scores used for patients with ACS will be discussed in this review using general performance metrics, model discrimination and calibration, and if available, results of both internal and external validation studies. An overview of all risk scores and pathways with their components are found in the Supplementary data (I–III).

## 2. Risk Scores Used at Presentation

The initial differentiation between non-ST-segment elevation ACS (NSTE-ACS), i.e., UA and NSTEMI, and other (benign) causes of chest pain is not always clear. Therefore, most practices perform adequate risk assessment based on objective parameters, clinical judgement and risk scores. The risk scores most commonly used for patients with suspected NSTE-ACS are found in Table 1 and Supplementary data I.

The history, electrocardiogram, age, risk factors, troponin (HEART)-score is a simple five-item risk score consisting of variables that were chosen based on clinical experience (“common sense”) and medical literature [7,8]. The HEART-score stratifies patients into low, moderate or high risk for developing major adverse cardiac events (MACE) up to six weeks after ACS and is also used as a decision aid to early discharge patients at low risk [9]. The HEART-score has shown to outperform risk scores such as the Global Registry of Acute Coronary Events (GRACE) and the Thrombolysis in Myocardial Infarction (TIMI) risk score for UA/NSTEMI in terms of the prediction of MACE [9], but has shown limitations since the score is based on older generation troponin testing and does not account for serial troponin measurements [10]. Attempts to modify the HEART-score complement the shortcomings of the original HEART-score and have shown good results in prospective trials [11,12].

Other risk scores or decision rules, such as the Emergency Department Assessment of Chest pain Score (EDACS) [13,14] or the Troponin-only Manchester Acute Coronary Syndromes (T-MACS) decision aids [15,16] have shown positive results and similar to even better performance than the HEART-score (Table 1). All of these scores show high sensitivity and negative predictive values (NPV) at the cost of specificity and positive predictive values (PPV) for their outcome, and all outperform a troponin-only strategy to rule-out ACS [17]. Combining these risk scores with serial troponin measurements (e.g., HEART pathway [11,18], A 2-h Accelerated Diagnostic Protocol to Assess Patients With Chest Pain Symptoms Using Contemporary Troponins as the Only Biomarker (ADAPT-ADP)) [19,20]) improves their performance and are also used in clinical practice as decision rules (Table 1). In fact, the European Society of Cardiology (ESC) guidelines for NSTE-ACS state that a rapid rule-out and rule-in protocol should be considered, if high-sensitivity troponins and a valid algorithm is available (class IIa, level of evidence (LoE) B) [5]. Numerous novel risk scores and accelerated diagnostic pathways have been developed and validated since the publication of these guidelines. The ESC Task Force on Chest Pain recommends the use of the HEART-score for patients with suspected ACS, stating that it most closely follows the clinical reasoning process in the diagnosis of acute chest pain [21]. However, it is still unclear which risk score or pathway might be considered best in ruling out low risk patients, and physicians are often inclined to practice their risk assessment strategy based on personal preference and clinical experience.

## 3. Risk Scores during ACS Admission

### 3.1. Mortality/Ischemic Risk

In both American and European guidelines, it is advised to use risk scores for the prognosis assessment for early risk stratification, i.e., ischemic and bleeding risk assessment at the start of hospital admission (Table 2) [2,5]. Both guidelines recommend the use of the GRACE score for this purpose [2,4,5]. Multiple GRACE risk models have been developed from the international GRACE registry [22,23], but the simplified GRACE risk score has been extensively evaluated and predominantly used in clinical practice [24,25]. The GRACE score estimates the in-hospital and the admission to six months risk for mortality alone and for mortality and MI combined. In addition, the updated GRACE 2.0 risk score predicts the one to three year risk for the same end points [26]. Compared to the TIMI risk score for UA/NSTEMI, the GRACE score has shown to be superior in terms of discrimination for predicting both in-hospital and long-term cardiovascular events [25]. Additionally, GRACE score outcome can guide the optimal timing of invasive strategy in patients with NSTE-ACS [27]. For example, patients with high mortality risk (GRACE score > 140) should undergo coronary angiography within 24 h of initial presentation [5]. The GRACE score for mortality from admission to six months is most often used for this end. Assessment of the GRACE score can also be used as a quality indicator for quality improvement initiatives [4], and GRACE-score guided strategies have also shown to reduce MACE rates compared to standard strategies [28].

Developed by the TIMI study group in the early 2000s, two separate risk scores were made from two different cohorts, the TIMI risk score for UA/NSTEMI predicted the risk of a combined end point of death, MI or urgent revascularization within two weeks after presentation and the TIMI risk score for STEMI predicted the 30 day mortality risk (Table 2) [29,30]. While outperformed by other risk scores in terms of predictive performance, the TIMI risk score for UA/NSTEMI has been modified into a validated clinical decision rule for patients with suspected NSTE-ACS (i.e., ADAPT-ADP) [19,25]. The TIMI risk score for STEMI shows overall similar performance as compared to the GRACE score; however, the use of the GRACE score might be favored in routine clinical practice due to its applicability across the entire spectrum of ACS [31].

From The Patterns of Nonadherence to Antiplatelet Regimens in Stented Patients (PARIS) registry [32], a prospective, multicentre, observational study of patients receiving percutaneous coronary intervention (PCI) with stent placement, the eponymous PARIS risk scores for major bleeding and for coronary thrombotic events (CTE) were created [33]. The PARIS CTE risk score predicts the risk of stent thrombosis and myocardial infarction for up to two years after PCI. The score showed acceptable results in both the derivation and validation cohort, but external validation studies showed limited to poor discrimination thus far [34,35]. As the simplicity of the CTE score might be favorable for clinical use, its value above other ischemic scores is yet to be established.

As the majority of ACS patients receive PCI or coronary artery bypass grafting, the residual ischemic risk in these patients is largely influenced by the complexity of the procedure [36]. Reassessment of residual ischemic risk before discharge may aid in choosing the optimal antithrombotic treatment strategy in terms of drug type, number of anticoagulant drugs (mono, dual or triple therapy) and duration of antiplatelet therapy. Intensified antithrombotic strategies should be considered in patients at high risk of recurrent MI or stent thrombosis but should always be weighed against individual bleeding risk.

### 3.2. Bleeding Risk

Similar to ischemic risk assessment, evaluation of bleeding risk should also be evaluated at initial hospital admission as recommended by the guidelines [5]. As the majority of ACS patients are discharged with dual antiplatelet therapy (DAPT), bleeding risk reassessment before discharge is often applied to guide DAPT duration. The importance of bleeding risk assessment is best characterized by the significant comorbidity and high rates of mortality in those who have suffered from major bleeding [37]. The most relevant risk scores used during ACS hospital admission are summarized in Table 2.

The Can Rapid Risk Stratification of Unstable Angina Pectoris Suppress Adverse Outcomes with Early Implementation of the ACC/AHA guidelines Quality Improvement Initiative (CRUSADE) was developed from a large retrospective database of high-risk patients with NSTE-ACS and estimates the patient’s likelihood of a major bleeding event during admission [38]. External validation showed acceptable to good discrimination for the prediction of in-hospital major bleeding and the score was overall well calibrated across different study populations [39,40,41]. The CRUSADE score outperformed other scores in terms of predicting in-hospital major bleeding events [40]. The ESC guideline on NSTE-ACS states that the use of the CRUSADE score may be considered in NSTE-ACS patients undergoing coronary angiography to quantify bleeding risk (class IIb, level of evidence B) [5,42]. A combined GRACE and CRUSADE-score strategy to guide the choice of P2Y12-inhibitor (clopidogrel vs. ticagrelor) significantly lowered the rates of MACE but without a difference in major bleeding [28]. An almost three-fold greater risk of major bleeding was found in patients with high CRUSADE scores when treated with 24 months versus six months DAPT (9.7% vs. 3.7%; *p* = 0.04), while no significant differences were found in those with low or intermediate bleeding risk [43]. These results may imply that the CRUSADE can also be used to guide DAPT duration.

The PARIS risk score for major bleeding was developed from the same previously mentioned PARIS registry, in which also patients on oral anticoagulation were included [33]. This six-item risk score showed reasonable, but also poor discrimination for major bleeding up to two years post-PCI across different validation cohorts [44,45,46]. Though external validation of both PARIS risk scores showed mixed results, combining these risk scores to estimate an ischemic-haemorrhagic balance allows physicians to choose the optimal antithrombotic strategy (e.g., duration of DAPT) after ACS and/or PCI.

The Predicting Bleeding Complications in Patients Undergoing Stent Implantation and Subsequent Dual AntiPlatelet Therapy (PRECISE-DAPT) risk score was developed from eight randomized controls trials (RCT) of PCI patients receiving DAPT, in which the majority of these RCTs patients were randomized to different DAPT durations (from 3 to 24 months) [47]. Five bleeding variables were identified as important predictors for major or minor bleeding at 12 months, but bleeding events to seven days post-PCI were excluded (Table 2). Overall, the PRECISE-DAPT score has shown acceptable discrimination for the prediction of major bleeding and was adequately calibrated across the validation cohorts [35,46,47,48]. Furthermore, the PRECISE-DAPT score was also evaluated in patients who were randomized to either ‘short’ (three or six months) or ‘longer’ (12 to 24 months) DAPT. This analysis showed that patients at high risk for bleeding with prolonged DAPT as compared to short DAPT had increased bleeding rates without any differences in ischemic events, while patients at low or moderate bleeding risk who have had longer DAPT showed similar bleeding rates and a significant reduction in ischemic events compared to those with short DAPT [47]. This effect was even more prominent in a subgroup of ACS patients with prolonged DAPT, with a number needed to harm of 38 in the high bleeding risk group, and a number needed to treat of 24 in patients without high bleeding risk. Therefore, the PRECISE-DAPT score is addressed in the ESC focused update on DAPT and the use of this score can be considered in determining optimal DAPT duration [3]. An important limitation of this score is that patients with an indication for oral anticoagulation (OAC) have been excluded from the derivation cohort, leaving uncertainty for its applicability in this subgroup.

Specifically designed for ACS patients, the (Bleeding Complications in a Multicenter Registry of Patients Discharged with Diagnosis of Acute Coronary Syndrome (BleeMACS) risk score was developed from a large international registry (over 15,000 patients) and validated in a nationwide Swedish registry (>90,000 patients), and identifies those patients at high risk for serious spontaneous bleeding post-discharge [49]. With promising results in terms of discrimination over the entire spectrum of ACS (including conservatively managed patients and patients with oral anticoagulation), this novel risk score might be also useful in tailoring antithrombotic therapy.

Multiple risk scores have been developed over the last decade, and a recently conducted meta-analysis on bleeding risk scores showed an aggregated c-statistic of 0.68 [95% confidence interval 0.65–0.72] [50]. While most risk scores are able to assess individual bleeding risk adequately, close to none have been prospectively validated to date as decision tools with the intent to reduce bleeding. This gap in current literature has previously been addressed in the guidelines, whereas ongoing trials might strengthen the evidence on the clinical value of these risk scores [3,51].

## 4. Long Term Risk Post-ACS

Patients with ACS have shown to be at higher risk for mortality, subsequent ischemic and bleeding events than patients with stable coronary artery disease (CAD). As risk is not static, it may be wise to consider reassessing the risks in the months or years following ACS. A risk score designed specifically to assess long term risk is the Dual AntiPlatelet Therapy (DAPT)-score (Table 3) [52]. The DAPT-score is one of few that combines individual ischemic and bleeding risk into one simple score. The score was developed from the randomized DAPT study [53], in which PCI patients after 12 months DAPT were randomized to either aspirin with a P2Y12-inhibitor or to aspirin with placebo for an additional 18 months. In this study, longer DAPT significantly reduced MI and stent thrombosis (ST) at the cost of major bleeding. From this cohort (n = 11,648), separate prediction models were created for MI or ST and for moderate or severe bleeding. Both internal and external validation showed moderate to acceptable discrimination for the separate models [52]. These models were combined to calculate the net clinical benefit (ischemic versus bleeding risk) and identify those who would benefit or be at harm from an extended DAPT duration. External validation of the final DAPT-score showed acceptable to poor discrimination for both bleeding and ischemic events, but conventional validation methods may not be suitable for this combined score [52,54]. In fact, a pooled meta-analysis of the DAPT-score showed that patients at relatively higher risk for ischemic events would benefit from extended DAPT duration without increased bleeding, and those at higher risk for bleeding showed no benefit in MI or ST rates and more bleeding events when DAPT was extended [55]. No specific sub-analyses have been performed on ACS patients; however, the majority of patients in the validation studies were ACS patients [55]. Moreover, given the recommended default DAPT duration of six months after elective PCI in chronic coronary syndromes (CCS), it is less likely that the DAPT-score will be applied in these patients [3]. Nevertheless, the ESC focused update on DAPT states that the DAPT-score can be considered to determine optimal DAPT duration, similar to the PRECISE-DAPT score.

Long-term risk scores for primary cardiovascular disease prevention, such as Systematic Coronary Risk Evaluation (SCORE), are widely used and recommended by prevention guidelines [56,57]. In the latest ESC guideline on CCS, secondary prevention measures should be taken according to individual risk of residual cardiac mortality in patients with established CCS [58]. High event risk is defined as a cardiac mortality rate of 3% or more per year, and low event risk as less than 1% per year, and can be assessed using different modalities (e.g., electrocardiogram stress testing, single photon emission computed tomography (SPECT) or cardiac magnetic resonance imaging (CMR)). However, these modalities are not all routinely performed in patients who have already endured ACS. In this guideline, risk score assessment is advised in a proposed algorithm for patients with CCS (both ‘stable’ CAD and post-ACS) and can be applied in the months and years following ACS. No specific risk scores are recommended, but a biomarker-based risk score (ABC-CHD) is mentioned with promising results [59]. Other high potential tools for long-term risk assessment are the Secondary Manifestations of ARTerial disease (SMART) and SMART-Reduction of Atherothrombosis for Continued Health (SMART-REACH) risk scores, which estimates the 10-year risk and lifetime risk of mortality in patients with established CAD [60,61,62]. A unique aspect of these tools is the option to include treatment options or lifestyle changes that alter your long-term risk for ischemic events. A visual assessment of the risk is made using graphs, making it accessible to the patient and is most suitable for shared decision-making.

## 5. Discussion

Risk scores used for patients with ACS are widely used and often available on online calculators. The most optimal risk score should be easily accessible, simple to interpret, adequately assess the risk of the outcome interested and provide treatment advice based on individually calculated risk. Its use should complement clinical judgement and contribute to primary or secondary prevention. It is implausible that one utopic risk model will become available for every risk and every situation, as it is also impractical to have separate risk scores for all risks and outcomes. The large heterogeneity (in demographic and clinical characteristics, definitions used for predictors and outcomes) in study cohorts in which risk scores or models are evaluated will in most cases explain why discrimination and calibration measures often perform less well in external validation studies. Nevertheless, even risk scores with moderate or limited discriminative values are recommended by international guidelines and used worldwide in clinical practice (e.g., CHA_2_DS_2_VASc risk score) [63].

If a risk score is used in clinical practice, it is important to know its specific limitations. For example, the PRECISE-DAPT and DAPT scores were developed from large PCI cohorts in patients treated with DAPT, but those with a long-term indication for OAC were excluded in these cohorts. These scores are also not validated in conservatively treated ACS patients, which make up for 10–30% of all NSTE-ACS patients [5]. Though it might still be helpful to calculate these scores for global risk estimation, it may not be wise to solely depend on score outcome in these specific patient groups. Apart from score limitations, it may be even more helpful to know the ‘strengths’ of a risk score and to know which score should be used in which situation. The most important risk scores and their strengths with level of evidence have been addressed by the ESC Guidelines.

**Figure 1:**
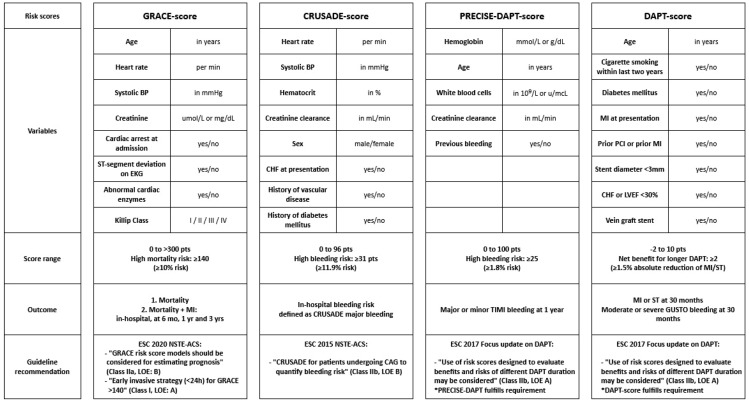
Risk scores endorsed by the guidelines of the European Society of Cardiology. Abbreviations: BP: blood pressure. CHF: congestive heart failure. DAPT: dual antiplatelet therapy. ESC: European Society of Cardiology. LVEF: left ventricular ejection fraction. LOE: level of evidence. MI: myocardial infarction. mo: months NSTE-ACS: non-ST elevation acute coronary syndrome. PCI: percutaneous coronary intervention. ST: stent thrombosis.

A short overview of risk scores recommended by the current guidelines for ACS patients are summarized in Figure 1. The GRACE score is currently the only risk score with a class IIa and class I recommendation in the NSTE-ACS guidelines. A possible hazard of GRACE score assessment is that physicians might not know which GRACE score should be used for the timing of early invasive strategy, as multiple GRACE score outcomes are provided by the online calculators. Additionally, these recommendations may change over time as the first RCTs investigating the GRACE score in NSTE-ACS patients are still ongoing [64,65]. The CRUSADE, PRECISE-DAPT and DAPT scores are all mentioned in the ESC guidelines with a class IIb recommendations, mainly due to the lack of prospective, randomized studies investigating these risk scores. Nevertheless, their simplicity allows physicians to easily and frequently apply them in clinical practice.

Alternatives to traditional risk scores have been proposed. The Academic Research Consortium (ARC) have published a consensus-based definition on high bleeding risk (ARC-HBR) criteria [66]. Rather than identifying these criteria using large datasets and regression analysis, the ARC-HBR criteria consist of variables or conditions known to cause an increased risk for bleeding based on previous literature. Thus far, the ARC-HBR-criteria adequately identify those at high bleeding risk in post-hoc analyses [46,67]. Furthermore, 35–40% of patients were identified as HBR, with relatively high sensitivity and NPV at the expense of specificity and PPV compared to other bleeding risk scores [46]. A recurrent issue in these post-hoc analyses is that multiple variables of the ARC-HBR criteria were either not available (e.g., chronic bleeding diathesis, liver cirrhosis with portal hypertension) or needed to be modified in the analyzed cohorts [46,67]. As we await prospective validation, it is still unclear whether the ARC-HBR criteria outperform current bleeding risk scores in terms of bleeding prediction and as clinical decision tools.

Another impressive alternative is the use of machine learning to create and validate models based on large datasets or even electronic hospital records. In over 11,000 patients with suspected MI, machine learning was applied to create a model with the variables age, sex, paired cardiac troponin I concentrations (including time and rate of change), which could stratify patients into low, moderate or intermediate risk for MI [68]. Excellent calibration and discrimination were found with reported c-statistics of 0.96, and outperformed the 0/3 and 0/1 h troponin decision rules in terms of sensitivity, specificity, NPV and PPV for MI. As machine learning is becoming increasingly more popular, it is very likely that such models will find their way into clinical practice. Nevertheless, clinicians should always rely on their own clinical judgement with the accompanying aid of risk scores or decision rules to support them in decision-making.

## 6. Conclusions

Risk scores can be of value in prognosis assessment and decision-making management in patients with ACS. Physicians should meticulously choose the optimal risk score per situation and at different points in time along the ACS pathway [9,52].

## Figures and Tables

**Table 1:** Risk scores used at presentation for suspected non-ST-segment elevation acute coronary syndrome (NSTE-ACS).

Risk Scores	Derivation Cohort								Validation Studies						
	Population (n)/Period	Outcome	Event Rate, n (%)		Sens. (95%CI)	Spec. (95%CI)	NPV (95%CI)	PPV (95%CI)	No. of Studies	Total Sample Size	% MACE in Low Risk	Sens. (95%CI)	Spec. (95%CI)	NPV (95%CI)	PPV (95%CI)
			Total	Low Risk											
HEART-score	n = 880 2006	MACE < 6 wks	158 (18.0)	3 (0.99)	98.1 (94.6–99.6)	41.6 (37.9–45.2)	99.0 (97.1–99.8)	26.9 (23.3–30.7)	16	n = 21,323	2.4	0.97 (0.94–0.98)	0.38 (0.33–0.43)	0.98 (0.97–0.99)	0.22 (0.18–0.27)
EDACS	n = 1974 2007–2010	MACE < 30 d	305 (15.5)	3 (0.98)	99.0 (96.6–99.7)	52.8 (50.6–55.0)	99.6	26.5	6	n = 6774	0.3–11.2	65.5 to 99.0	23.8 to 62.6	88.9 to 99.7	16.6 to 18.7
T-MACS	n = 703 2006–2007	MACE < 30 d	157 (22.3)	2 (0.28)	98.7 (95.3–99.8)	47.6 (43.4–51.9)	99.3 (97.3–99.9)	34.0 (29.6–38.7)	2	n = 1864	0.7–0.7	95.2 to 100	44.4 to 47.0	99.3 to 100	23.9 to 29.1
**Risk score pathways**															
HEART pathway	n = 1070 * 2006–2007	MACE < 30 d	12 (1.1)	0 (0.00)	100 (72–100)	83.1 (81–85)	100 (99,100)	6.3 (3.5–11)	7	n = 12,844	0.0–1.2	92.8 to 100	38.7 to 53.5	98.3 to 100	10.7 to 27.6
EDACS-ADP	n = 1974 2007–2010	MACE < 30 d	305 (15.5)	3 (0.98)	99.0 (96.6–99.7)	49.9 (47.4–52.3)	99.6	26.5	6	n = 9136	0.0–1.1	88.2 to 100	26.2 to 70.2	98.9 to 100	16.0 to 35.2
ADAPT-ADP	n = 1975 2007–2011	MACE < 30 d	302 (15.3)	1 (0.25)	99.7 (98.1–99.1)	23.4 (21.4–25.5)	99.7 (98.6–100)	19.0 (17.2–21.0)	5	n = 5808	0.0–0.9	92.8 to 100	4.8 to 69.1	99.1 to 100	7.5 to 23.0

* Low risk for ACS: TIMI-score < 2. Diagnostic performance results are provided for risk scores only and risk score pathways for major adverse cardiac events (MACE) within 30 days or six weeks. Results from the derivation cohorts and validation cohorts are displayed separately. Results from the systematic review from Laureano et al. are used for the validation studies of the history, electrocardiogram, age, risk factors, troponin- (HEART)-score. Lowest and highest reported values measured in validation studies are reported of all other risk scores. All articles used in Table 1 can be found in the Supplementary data. In all studies, MACE was defined as the composite of death, myocardial infarction or revascularization. Abbreviations: ACS: acute coronary syndrome. ADP: accelerated diagnostic pathway. D: days. MACE: major adverse cardiac events. NPV: negative predictive value. NSTE-ACS: non-ST-elevation acute coronary syndrome. Sens.: sensitivity. Spec.: specificity. PPV: positive predictive value. Wks: weeks.

**Table 2:** Risk scores used during admission for patients with ACS.

Risk Score	Derivation Cohort						Validation Studies				
	Population (n)	ACS (%)	Period	Outcome	AUC (95%CI)	Calibration	No of Studies	Total Sample Size	ACS (%)	Events (%)	Reported AUCs
**Mortality/Ischemic Risk—During Admission**											
TIMI (UA/NSTEMI)	UA/NSTEMI (n = 1957)	100	1996–1998	Death, MI or urgent revascularisation < 14 d	0.65 [NA]	HL(p): 0.89	19	n = 27,986	11.1–100.0	5.4–42.5	0.59 to 0.88
TIMI (STEMI)	STEMI (n = 14,114)	100	1997–1998	Death ≤ 30 d	0.78 [NA]	CC: 0.99	16	n = 96,151	100	3.6–32.0	0.62 to 0.87
GRACE	ACS (n = 13,708)	100	1999–2001	In-hospital death	0.83 [NA]	VA: Good, HL(p): 0.52	17	n = 84,655	100	2.4–18.5	0.64 to 0.95
	ACS (n = 43,810)	100	1999–2005	Death ≤ 6 mo	0.82 [NA]	HL(p): 0.89	7	n = 20,676	100	6.4–11.2	0.66 to 0.85
PARIS CTE	PCI + DAPT (n = 4190)	37.8	2009–2010	MI + ST	0.70 [NA]	VA: Adequate	6	n = 35,736	51.7–100	1.0–3.4	0.57 to 0.65
**Bleeding Risk—During Admission**											
CRUSADE	NSTE-ACS (n = 71,277)	100	2003–2006	In-hospital bleeding	0.72 [NA]	NA	8	n = 31,365	100.0	2.1–9.8	0.71 to 0.88
PARIS MB	PCI + DAPT (n = 4190)	37.8	2009–2010	Non-CABG related major bleeding ≤ 2 yrs	0.72 [NA]	VA: Adequate	7	n = 35,775	44.1–100	0.5–4.0	0.56 to 0.74
PRECISE-DAPT	PCI + DAPT (n = 1963)	55.6	2007–2014	Major bleeding ≤ 1yr *	0.71 (0.57–0.85)	VA: Adequate	6	n = 34,692	34.4–100	1.0-–7.0	0.65 to 0.81
				Minor or major bleeding ≤ 1yr *	0.73 (0.61–0.85)		3	n = 17,735	34.4–100	1.5–13.2	0.61 to 0.70
BLEEMACS	ACS (n = 15,401)	100	2003–2014	Serious bleed ≤ 1 yr	0.71 (0.68–0.74)	VA: Excellent	2	n > 94,000	100	4.0	0.65 to 0.77

Predictive performances are provided for scores predicting mortality or ischemic risk and for bleeding risk. Results from the derivation cohorts and validation cohorts are displayed separately. The lowest and highest reported discrimination values measured in validation studies are reported. Only those studies that validated the same (or similar) outcome are taken into account. All articles used in Table 2 can be found in the Supplementary data. Abbreviations: ACS: acute coronary syndrome. AUC: area under the receiver operating characteristic (ROC) curve. CABG: coronary artery bypass grafting. CC: correlation coefficient. DAPT: dual antiplatelet therapy. HL(p): *p*-value of the Hosmer–Lemeshow test. MI: myocardial infarction. Mo: months. NSTE-ACS: non-ST-elevation acute coronary syndrome. PCI: percutaneous coronary intervention. ROC: receiver operating characteristic. STEMI: ST-elevation MI. VA: visual assessment. Yr: year. *: Bleeding at 7 days post-PCI or later.

**Table 3:** Risk scores used post-ACS.

Risk Scores Used Post-ACS											
Risk Score	Derivation Cohort						Validation Studies				
	Population (n)	ACS (%)	Period	Prediction Model	AUC (95%CI)	Calibration	No of Studies	Total Sample Size	ACS (%)	Events (%)	Reported AUCs
DAPT SCORE—Ischemic model	N = 11,648	73.8	2009–2014	Definite/probable ST or MI at 30 mo	0.70 (0.68–0.73)	ND: 0.81	2	N = 49,237	44.7–64.7	1.0–3.1	0.64 to 0.67
DAPT SCORE—Bleeding model				Moderate or severe bleeding at 30 mo	0.68 (0.65–0.72)	ND: 0.34	2	N = 49,237	44.7–64.7	0.7–1.8	0.64 to 0.67
DAPT SCORE—Final risk score				Ischemic end point *	NA	NA	6	N = 61,263	40.1–100	1.3–17.0 *	0.53 to 0.71
				Bleeding end point *	NA	NA	6	N = 61,263	40.1–100	0.5–4.8 *	0.46 to 0.79

Predictive performances for the DAPT-score are shown for the ischemic model, bleeding model and the final risk score as a whole. Results from the derivation cohorts and validation cohorts are displayed separately. The lowest and highest reported discrimination values measured in validation studies are reported. Only those studies that validated the same (or similar) outcome are taken into account. All articles used in Table 3 can be found in the Supplementary data. Abbreviations: ACS: acute coronary syndrome. AUC: area under the receiver operating characteristic curve. DAPT: dual antiplatelet therapy. MI: myocardial infarction. mo: months. ND: Nam and D’Agostino. NSTE-ACS: non-ST-elevation acute coronary syndrome. PCI: percutaneous coronary intervention. STEMI: ST-elevation MI. * Different end point definitions are used across the validation studies.

## Supplementary Materials

The following are available online at https://www.mdpi.com/2077-0383/9/9/3039/s1, Supplementary data I: Risk scores/pathways for suspected NSTE-ACS, Supplementary data II: Risk scores during ACS admission, Supplementary data III: Risk scores used post-ACS, Supplementary data IV: References used in Tables S1–S3.

## References

[1] Yeh R.W., Sidney S., Chandra M., Sorel M., Selby J.V., Go A.S. (2010). Population trends in the incidence and outcomes of acute myocardial infarction. N. Engl. J. Med..

[2] Amsterdam E.A., Wenger N.K., Brindis R.G., Casey D.E.J., Ganiats T.G., Holmes D.R.J., Jaffe A.S., Jneid H., Kelly R.F., Kontos M.C. (2014). 2014 AHA/ACC guideline for the management of patients with non-ST-elevation acute coronary syndromes: A report of the American College of Cardiology/American Heart Association Task Force on Practice Guidelines. Circulation.

[3] Valgimigli M., Bueno H., Byrne R.A., Collet J.-P., Costa F., Jeppsson A., Juni P., Kastrati A., Kolh P., Mauri L. (2018). 2017 ESC focused update on dual antiplatelet therapy in coronary artery disease developed in collaboration with EACTS: The Task Force for dual antiplatelet therapy in coronary artery disease of the European Society of Cardiology (ESC) and of the European. Eur. Heart J..

[4] Ibanez B., James S., Agewall S., Antunes M.J., Bucciarelli-Ducci C., Bueno H., Caforio A.L.P., Crea F., Goudevenos J.A., Halvorsen S. (2018). 2017 ESC Guidelines for the management of acute myocardial infarction in patients presenting with ST-segment elevation: The Task Force for the management of acute myocardial infarction in patients presenting with ST-segment elevation of the European Socie. Eur. Heart J..

[5] Collet J.-P., Thiele H., Barbato E., Barthélémy O., Bauersachs J., Bhatt D.L., Dendale P., Dorobantu M., Edvardsen T., Folliguet T. (2020). 2020 ESC Guidelines for the management of acute coronary syndromes in patients presenting without persistent ST-segment elevation. Eur. Heart J..

[6] Kannel W.B., McGee D., Gordon T. (1976). A general cardiovascular risk profile: The Framingham study. Am. J. Cardiol..

[7] Six A.J., Backus B.E., Kelder J.C. (2008). Heart Score Value. Neth. Heart J..

[8] Backus B.E., Six A.J., Kelder J.C., Mast T.P., van den Akker F., Mast E.G., Monnink S.H.J., van Tooren R.M., Doevendans P.A.F.M. (2010). Chest pain in the emergency room: A multicenter validation of the HEART Score. Crit. Pathw. Cardiol..

[9] Backus B.E., Six A.J., Kelder J.C., Bosschaert M.A.R., Mast E.G., Mosterd A., Veldkamp R.F., Wardeh A.J., Tio R., Braam R. (2013). A prospective validation of the HEART score for chest pain patients at the emergency department. Int. J. Cardiol..

[10] Van Den Berg P., Body R. (2018). The HEART score for early rule out of acute coronary syndromes in the emergency department: A systematic review and meta-analysis. Eur. Heart J. Acute Cardiovasc. Care.

[11] Mahler S.A., Lenoir K.M., Wells B.J., Burke G.L., Duncan P.W., Case L.D., Herrington D.M., Diaz-Garelli J.F., Futrell W.M., Hiestand B.C. (2018). Safely Identifying Emergency Department Patients With Acute Chest Pain for Early Discharge. Circulation.

[12] Laureano-Phillips J., Robinson R.D., Aryal S., Blair S., Wilson D., Boyd K., Schrader C.D., Zenarosa N.R., Wang H. (2019). HEART Score Risk Stratification of Low-Risk Chest Pain Patients in the Emergency Department: A Systematic Review and Meta-Analysis. Ann. Emerg. Med..

[13] Than M., Flaws D., Sanders S., Doust J., Glasziou P., Kline J., Aldous S., Troughton R., Reid C., Parsonage W.A. (2014). Development and validation of the Emergency Department Assessment of Chest pain Score and 2 h accelerated diagnostic protocol. Emerg. Med. Australas..

[14] Mark D.G., Huang J., Chettipally U., Kene M.V., Anderson M.L., Hess E.P., Ballard D.W., Vinson D.R., Reed M.E. (2018). Performance of Coronary Risk Scores Among Patients with Chest Pain in the Emergency Department. J. Am. Coll. Cardiol..

[15] Body R., Carlton E., Sperrin M., Lewis P.S., Burrows G., Carley S., McDowell G., Buchan I., Greaves K., Mackway-Jones K. (2017). Troponin-only Manchester Acute Coronary Syndromes (T-MACS) decision aid: Single biomarker re-derivation and external validation in three cohorts. Emerg. Med. J..

[16] Body R., Morris N., Reynard C., Collinson P.O. (2020). Comparison of four decision aids for the early diagnosis of acute coronary syndromes in the emergency department. Emerg. Med. J..

[17] Chapman A.R., Hesse K., Andrews J., Ken Lee K., Anand A., Shah A.S.V., Sandeman D., Ferry A.V., Jameson J., Piya S. (2018). High-Sensitivity Cardiac Troponin I and Clinical Risk Scores in Patients With Suspected Acute Coronary Syndrome. Circulation.

[18] Mahler S.A., Hiestand B.C., Goff D.C.J., Hoekstra J.W., Miller C.D. (2011). Can the HEART score safely reduce stress testing and cardiac imaging in patients at low risk for major adverse cardiac events?. Crit. Pathw. Cardiol..

[19] Than M., Cullen L., Aldous S., Parsonage W.A., Reid C.M., Greenslade J., Flaws D., Hammett C.J., Beam D.M., Ardagh M.W. (2012). 2-Hour accelerated diagnostic protocol to assess patients with chest pain symptoms using contemporary troponins as the only biomarker: The ADAPT trial. J. Am. Coll. Cardiol..

[20] Cullen L., Mueller C., Parsonage W.A., Wildi K., Greenslade J.H., Twerenbold R., Aldous S., Meller B., Tate J.R., Reichlin T. (2013). Validation of high-sensitivity troponin I in a 2-h diagnostic strategy to assess 30-day outcomes in emergency department patients with possible acute coronary syndrome. J. Am. Coll. Cardiol..

[21] Stepinska J., Lettino M., Ahrens I., Bueno H., Garcia-Castrillo L., Khoury A., Lancellotti P., Mueller C., Muenzel T., Oleksiak A. (2020). Diagnosis and risk stratification of chest pain patients in the emergency department: Focus on acute coronary syndromes. A position paper of the Acute Cardiovascular Care Association. Eur. Heart J. Acute Cardiovasc. Care.

[22] Granger C.B., Goldberg R.J., Dabbous O., Pieper K.S., Eagle K.A., Cannon C.P., Van De Werf F., Avezum A., Goodman S.G., Flather M.D. (2003). Predictors of hospital mortality in the global registry of acute coronary events. Arch. Intern. Med..

[23] Eagle K.A., Lim M.J., Dabbous O.H., Pieper K.S., Goldberg R.J., Van de Werf F., Goodman S.G., Granger C.B., Steg P.G., Gore J.M. (2004). A validated prediction model for all forms of acute coronary syndrome: Estimating the risk of 6-month postdischarge death in an international registry. JAMA.

[24] Fox K.A.A., Dabbous O.H., Goldberg R.J., Pieper K.S., Eagle K.A., Van De Werf F., Avezum Á., Goodman S.G., Flather M.D., Anderson F.A. (2006). Prediction of risk of death and myocardial infarction in the six months after presentation with acute coronary syndrome: Prospective multinational observational study (GRACE). Br. Med. J..

[25] D’Ascenzo F., Biondi-Zoccai G., Moretti C., Bollati M., Omedè P., Sciuto F., Presutti D.G., Modena M.G., Gasparini M., Reed M.J. (2012). TIMI, GRACE and alternative risk scores in Acute Coronary Syndromes: A meta-analysis of 40 derivation studies on 216,552 patients and of 42 validation studies on 31,625 patients. Contemp. Clin. Trials.

[26] Fox K.A.A., FitzGerald G., Puymirat E., Huang W., Carruthers K., Simon T., Coste P., Monsegu J., Steg P.G., Danchin N. (2014). Should patients with acute coronary disease be stratified for management according to their risk? Derivation, external validation and outcomes using the updated GRACE risk score. BMJ Open.

[27] Mehta S.R., Granger C.B., Boden W.E., Steg P.G., Bassand J.-P., Faxon D.P., Afzal R., Chrolavicius S., Jolly S.S., Widimsky P. (2009). Early versus delayed invasive intervention in acute coronary syndromes. N. Engl. J. Med..

[28] Antoniou S., Colicchia M., Guttmann O.P., Rathod K.S., Wright P., Fhadil S., Knight C.J., Jain A.K., Smith E.J., Mathur A. (2018). Risk scoring to guide antiplatelet therapy post-percutaneous coronary intervention for acute coronary syndrome results in improved clinical outcomes. Eur. Heart J. Qual. Care Clin. Outcomes.

[29] Antman E.M., Cohen M., Bernink P.J.L.M., McCabe C.H., Horacek T., Papuchis G., Mautner B., Corbalan R., Radley D., Braunwald E. (2000). The TIMI risk score for unstable angina/non-ST elevation MI: A method for prognostication and therapeutic decision making. J. Am. Med. Assoc..

[30] Morrow D.A., Antman E.M., Charlesworth A., Cairns R., Murphy S.A., de Lemos J.A., Giugliano R.P., McCabe C.H., Braunwald E. (2000). TIMI risk score for ST-elevation myocardial infarction: A convenient, bedside, clinical score for risk assessment at presentation: An intravenous nPA for treatment of infarcting myocardium early II trial substudy. Circulation.

[31] Méndez-Eirín E., Flores-Ríos X., García-López F., Pérez-Pérez A.J., Estévez-Loureiro R., Piñón-Esteban P., Aldama-López G., Salgado-Fernández J., Calviño-Santos R.A., Vázquez Rodríguez J.M. (2012). Comparison of the prognostic predictive value of the TIMI, PAMI, CADILLAC, and GRACE risk scores in STEACS undergoing primary or rescue PCI. Rev. Esp. Cardiol. (Engl. Ed.).

[32] Mehran R., Baber U., Steg P.G., Ariti C., Weisz G., Witzenbichler B., Henry T.D., Kini A.S., Stuckey T., Cohen D.J. (2013). Cessation of dual antiplatelet treatment and cardiac events after percutaneous coronary intervention (PARIS): 2 year results from a prospective observational study. Lancet.

[33] Baber U., Mehran R., Giustino G., Cohen D.J., Henry T.D., Sartori S., Ariti C., Litherland C., Dangas G., Michael Gibson C. (2016). Coronary Thrombosis and Major Bleeding after PCI with Drug-Eluting Stents Risk Scores from Paris. J. Am. Coll. Cardiol..

[34] Song L., Guan C., Yan H., Qiao S., Wu Y., Yuan J., Dou K., Yang Y., Dangas G.D., Xu B. (2018). Validation of contemporary risk scores in predicting coronary thrombotic events and major bleeding in patients with acute coronary syndrome after drug-eluting stent implantations. Catheter. Cardiovasc. Interv..

[35] Bianco M., D’ascenzo F., Raposeiras Roubin S., Kinnaird T., Peyracchia M., Ariza-Sole A., Cerrato E., Manzano-Fernandez S., Gravinese C., Templin C. (2020). Comparative external validation of the PRECISE-DAPT and PARIS risk scores in 4424 acute coronary syndrome patients treated with prasugrel or ticagrelor. Int. J. Cardiol..

[36] Giustino G., Chieffo A., Palmerini T., Valgimigli M., Feres F., Abizaid A., Costa R.A., Hong M.-K., Kim B.-K., Jang Y. (2016). Efficacy and Safety of Dual Antiplatelet Therapy After Complex PCI. J. Am. Coll. Cardiol..

[37] Valgimigli M., Costa F., Lokhnygina Y., Clare R.M., Wallentin L., Moliterno D.J., Armstrong P.W., White H.D., Held C., Aylward P.E. (2017). Trade-off of myocardial infarction vs. bleeding types on mortality after acute coronary syndrome: Lessons from the Thrombin Receptor Antagonist for Clinical Event Reduction in Acute Coronary Syndrome (TRACER) randomized trial. Eur. Heart J..

[38] Subherwal S., Bach R.G., Chen A.Y., Gage B.F., Rao S.V., Newby L.K., Wang T.Y., Gibler W.B., Ohman E.M., Roe M.T. (2009). Baseline risk of major bleeding in non-sT-segment- elevation myocardial infarction the CRUSADE (can rapid risk stratification of unstable angina patients suppress ADverse outcomes with early implementation of the ACC/AHA guidelines) bleeding score. Circulation.

[39] Abu-Assi E., Gracía-Acuña J.M., Ferreira-González I., Peña-Gil C., Gayoso-Diz P., González-Juanatey J.R. (2010). Evaluating the Performance of the Can Rapid Risk Stratification of Unstable Angina Patients Suppress Adverse Outcomes With Early Implementation of the ACC/AHA Guidelines (CRUSADE) bleeding score in a contemporary Spanish cohort of patients with non-ST-se. Circulation.

[40] Abu-Assi E., Raposeiras-Roubin S., Lear P., Cabanas-Grandío P., Girondo M., Rodríguez-Cordero M., Pereira-López E., Romaní S.G., González-Cambeiro C., Alvarez-Alvarez B. (2012). Comparing the predictive validity of three contemporary bleeding risk scores in acute coronary syndrome. Eur. Heart J. Acute Cardiovasc. Care.

[41] Flores-Ríos X., Couto-Mallón D., Rodríguez-Garrido J., García-Guimaraes M., Gargallo-Fernández P., Piñón-Esteban P., Aldama-López G., Salgado-Fernández J., Calviño-Santos R., Vázquez-González N. (2013). Comparison of the performance of the CRUSADE, ACUITY-HORIZONS, and ACTION bleeding risk scores in STEMI undergoing primary PCI: Insights from a cohort of 1391 patients. Eur. Heart J. Acute Cardiovasc. Care.

[42] Roffi M., Patrono C., Collet J.P., Mueller C., Valgimigli M., Andreotti F., Bax J.J., Borger M.A., Brotons C., Chew D.P. (2016). 2015 ESC Guidelines for the management of acute coronary syndromes in patients presenting without persistent st-segment elevation: Task force for the management of acute coronary syndromes in patients presenting without persistent ST-segment elevation of. Eur. Heart J..

[43] Costa F., Tijssen J.G., Ariotti S., Giatti S., Moscarella E., Guastaroba P., De Palma R., Andò G., Oreto G., Zijlstra F. (2015). Incremental value of the CRUSADE, ACUITY, and HAS-BLED risk scores for the prediction of hemorrhagic events after coronary stent implantation in patients undergoing long or short duration of dual antiplatelet therapy. J. Am. Heart Assoc..

[44] Abu-Assi E., Raposeiras-Roubin S., Cobas-Paz R., Caneiro-Queija B., Martínez-Reglero C., Rodríguez-Rodríguez J.M., Baz A., Íñiguez-Romo A. (2018). Assessing the performance of the PRECISE-DAPT and Paris risk scores for predicting one-year out-of-hospital bleeding in acute coronary syndrome patients. EuroIntervention.

[45] Raposeiras-Roubín S., Caneiro Queija B., D’Ascenzo F., Kinnaird T., Ariza-Solé A., Manzano-Fernández S., Templin C., Velicki L., Xanthopoulou I., Cerrato E. (2019). Usefulness of the PARIS Score to Evaluate the Ischemic-hemorrhagic Net Benefit With Ticagrelor and Prasugrel After an Acute Coronary Syndrome. Rev. Esp. Cardiol..

[46] Ueki Y., Bar S., Losdat S., Otsuka T., Zanchin C., Zanchin T., Gragnano F., Gargiulo G., Siontis G.C.M., Praz F. (2020). Validation of Bleeding Risk Criteria (ARC-HBR) in Patients Undergoing Percutaneous Coronary Intervention and Comparison with Contemporary Bleeding Risk Scores. EuroInterv. J. Eur. Collab. Work. Gr. Interv. Cardiol. Eur. Soc. Cardiol..

[47] Costa F., van Klaveren D., James S., Heg D., Räber L., Feres F., Pilgrim T., Hong M.K., Kim H.S., Colombo A. (2017). Derivation and validation of the predicting bleeding complications in patients undergoing stent implantation and subsequent dual antiplatelet therapy (PRECISE-DAPT) score: A pooled analysis of individual-patient datasets from clinical trials. Lancet.

[48] Choi S.Y., Kim M.H., Cho Y.R., Sung Park J., Min Lee K., Park T.H., Yun S.C. (2018). Performance of PRECISE-DAPT Score for Predicting Bleeding Complication During Dual Antiplatelet Therapy. Circ. Cardiovasc. Interv..

[49] Raposeiras-Roubín S., Faxén J., Íñiguez-Romo A., Henriques J.P.S., D’Ascenzo F., Saucedo J., Szummer K., Jernberg T., James S.K., Juanatey J.R.G. (2018). Development and external validation of a post-discharge bleeding risk score in patients with acute coronary syndrome: The BleeMACS score. Int. J. Cardiol..

[50] Ko S.Q., Valsdottir L.R., Strom J.B., Cheng Y.C., Hirayama A., Liu P.H., Yanagisawa N., Yen H., Shen C., Yeh R.W. (2018). Meta-Analysis of Bleeding Risk Prediction Scores in Patients After Percutaneous Coronary Intervention on Dual Antiplatelet Therapy. Am. J. Cardiol..

[51] Frigoli E., Smits P., Vranckx P., Ozaki Y., Tijssen J., Juni P., Morice M.-C., Onuma Y., Windecker S., Frenk A. (2019). Design and rationale of the Management of High Bleeding Risk Patients Post Bioresorbable Polymer Coated Stent Implantation With an Abbreviated Versus Standard DAPT Regimen (MASTER DAPT) Study. Am. Heart J..

[52] Yeh R.W., Secemsky E.A., Kereiakes D.J., Normand S.L.T., Gershlick A.H., Cohen D.J., Spertus J.A., Steg P.G., Cutlip D.E., Rinaldi M.J. (2016). Development and validation of a prediction rule for benefit and harm of Dual antiplatelet therapy beyond 1 year after percutaneous coronary intervention. JAMA—J. Am. Med. Assoc..

[53] Mauri L., Kereiakes D.J., Yeh R.W., Driscoll-Shempp P., Cutlip D.E., Steg P.G., Normand S.L.T., Braunwald E., Wiviott S.D., Cohen D.J. (2014). Twelve or 30 months of dual antiplatelet therapy after drug-eluting stents. N. Engl. J. Med..

[54] Ueda P., Jernberg T., James S., Alfredsson J., Erlinge D., Omerovic E., Persson J., Ravn-Fischer A., Tornvall P., Svennblad B. (2018). External Validation of the DAPT Score in a Nationwide Population. J. Am. Coll. Cardiol..

[55] Witberg G., Zusman O., Yahav D., Perl L., Vaknin-Assa H., Kornowski R. (2019). Meta-Analysis of Studies Examining the External Validity of the DAPT Score. Eur. Heart J. Cardiovasc. Pharmacother..

[56] Conroy R.M., Pyörälä K., Fitzgerald A.P., Sans S., Menotti A., De Backer G., De Bacquer D., Ducimetière P., Jousilahti P., Keil U. (2003). Estimation of ten-year risk of fatal cardiovascular disease in Europe: The SCORE project. Eur. Heart J..

[57] Piepoli M.F., Hoes A.W., Agewall S., Albus C., Brotons C., Catapano A.L., Cooney M.-T., Corrà U., Cosyns B., Deaton C. (2016). 2016 European Guidelines on cardiovascular disease prevention in clinical practice: The Sixth Joint Task Force of the European Society of Cardiology and Other Societies on Cardiovascular Disease Prevention in Clinical Practice (constituted by representat. Eur. Heart J..

[58] Knuuti J., Wijns W., Saraste A., Capodanno D., Barbato E., Funck-Brentano C., Prescott E., Storey R.F., Deaton C., Cuisset T. (2020). 2019 ESC Guidelines for the diagnosis and management of chronic coronary syndromes. Eur. Heart J..

[59] Lindholm D., Lindbäck J., Armstrong P.W., Budaj A., Cannon C.P., Granger C.B., Hagström E., Held C., Koenig W., Östlund O. (2017). Biomarker-Based Risk Model to Predict Cardiovascular Mortality in Patients With Stable Coronary Disease. J. Am. Coll. Cardiol..

[60] Dorresteijn J.A.N., Visseren F.L.J., Wassink A.M.J., Gondrie M.J.A., Steyerberg E.W., Ridker P.M., Cook N.R., van der Graaf Y. (2013). Development and validation of a prediction rule for recurrent vascular events based on a cohort study of patients with arterial disease: The SMART risk score. Heart.

[61] Kaasenbrood L., Boekholdt S.M., van der Graaf Y., Ray K.K., Peters R.J.G., Kastelein J.J.P., Amarenco P., LaRosa J.C., Cramer M.J.M., Westerink J. (2016). Distribution of Estimated 10-Year Risk of Recurrent Vascular Events and Residual Risk in a Secondary Prevention Population. Circulation.

[62] Kaasenbrood L., Bhatt D.L., Dorresteijn J.A.N., Wilson P.W.F., D’Agostino R.B., Massaro J.M., van der Graaf Y., Cramer M.J.M., Kappelle L.J., de Borst G.J. (2018). Estimated life expectancy without recurrent cardiovascular events in patients with vascular disease: The SMART-REACH model. J. Am. Heart Assoc..

[63] Kirchhof P., Benussi S., Kotecha D., Ahlsson A., Atar D., Casadei B., Castella M., Diener H.C., Heidbuchel H., Hendriks J. (2016). 2016 ESC Guidelines for the management of atrial fibrillation developed in collaboration with EACTS. Europace.

[64] Chew D.P., Astley C.M., Luker H., Alprandi-Costa B., Hillis G., Chow C.K., Quinn S., Yan A.T., Gale C.P., Goodman S. (2015). A cluster randomized trial of objective risk assessment versus standard care for acute coronary syndromes: Rationale and design of the Australian GRACE Risk score Intervention Study (AGRIS). Am. Heart J..

[65] Everett C.C., Fox K.A., Reynolds C., Fernandez C., Sharples L., Stocken D.D., Carruthers K., Hemingway H., Yan A.T., Goodman S.G. (2019). Evaluation of the impact of the GRACE risk score on the management and outcome of patients hospitalised with non-ST elevation acute coronary syndrome in the UK: Protocol of the UKGRIS cluster-randomised registry-based trial. BMJ Open.

[66] Urban P., Mehran R., Colleran R., Angiolillo D.J., Byrne R.A., Capodanno D., Cuisset T., Cutlip D., Eerdmans P., Eikelboom J. (2019). Defining high bleeding risk in patients undergoing percutaneous coronary intervention: A consensus document from the Academic Research Consortium for High Bleeding Risk. Eur. Heart J..

[67] Natsuaki M., Morimoto T., Shiomi H., Yamaji K., Watanabe H., Shizuta S., Kato T., Ando K., Nakagawa Y., Furukawa Y. (2019). Application of the Academic Research Consortium High Bleeding Risk Criteria in an All-Comers Registry of Percutaneous Coronary Intervention. Circ. Cardiovasc. Interv..

[68] Than M.P., Pickering J.W., Sandoval Y., Shah A.S.V., Tsanas A., Apple F.S., Blankenberg S., Cullen L., Mueller C., Neumann J.T. (2019). Machine Learning to Predict the Likelihood of Acute Myocardial Infarction. Circulation.

