# Supplementary material for: Risk Assessment Using Risk Scores in Patients with Acute Coronary Syndrome
Source: J Clin Med. 2020 Sep 21;9(9):3039. doi: 10.3390/jcm9093039 (PMC7565031; doi:10.3390/jcm9093039)
Supplement: Supplementary file 1 [file jcm-09-03039-s001.pdf]

Supplementary Materials

I. Risk scores / pathways for suspected NSTE-ACS

a. HEART-score / HEART pathway

b. EDACS / EDACS-ADP

c. T-MACS

d. ADAPT-ADP

II. Risk scores during ACS admission

Mortality / ischemic risk

a. GRACE score

b. TIMI risk score for UA/NSTEMI

c. TIMI risk score for STEMI

d. PARIS CTE score

Bleeding risk

e. CRUSADE score

f. PARIS MB score

g. PRECISE-DAPT score

h. BleeMACS score

III. Risk scores used post-ACS

a. DAPT score

IV. References used in Tables 1-3.

I. Risk scores / pathways for suspected NSTE-ACS

a. HEART-score[1,2] / HEART pathway[3,4]

Variables	Outcome	Score
History	Highly suspicious	2
	Moderately suspicious	1
	Slightly suspicious	0
ECG	Significant ST depression	2
	Nonspecific repolarization disturbance	1
	Normal	0
Age	≤65 year	2
	45-65 year	1
	≤45 year	0
Risk factors	≥ 3 risk factors or history of atherosclerotic disease	2
	1 or 2 risk factors	1
	No risk factors known	0
Troponin	>2x normal limit	2
	1-2x normal limit	1
	≤ normal limit	0

Supplementary Table 1a. Composition of the HEART score for chest pain patients in the emergency room.

Supplementary Table 1b. HEART score outcome and recommendations*

Total points	Risk of MACE**	Recommendation
0-3	Low (2.5%)	Discharge
4-6	Intermediate (20.3%)	Admission for clinical observation
≥7	High (72.7%)	Early invasive strategy

*All data is derived from the reference article Six et al.[1]

**Major adverse cardiac event (MACE) was defined as acute myocardial infarction, percutaneous coronary intervention, coronary artery bypass grafting and death.

Supplementary Table 2. HEART pathway and recommendations.[3,4] The original HEART score is used in this pathway, but the recommendations are dependent on the outcome of the (repeated) troponin values.

HEART Score	Initial troponin	Risk of 30-day MACE	Recommendation
≤3	0 points	Low (0.9-1.7%)	Repeat troponin at 3 hours and if negative, discharge home with outpatient follow-up.
	1-2 points	High (12-65%)	Cardiology consultation and admission recommended. Further testing indicated.
≥4	0 points	High (12-65%)	Admit to hospital or observation. Further testing indicated.
	1-2 points	High (12-65%)	Cardiology consultation and admission recommended. Further testing indicated.

b. EDACS / EDACS-ADP[5]

Supplementary Table 3a. Emergency department assessment of chest pain score (EDACS)

**Variables**	**Outcome**	**Score**
a. Age	18-45	+2
	46-50	+4
	51-55	+6
	56-60	+8
	61-65	+10
	66-70	+12
	71-75	+14
	76-80	+16
	81-85	+18
	86+	+20
b. Male sex		+6
c. Aged 18-50 years and either:<br>Known coronary artery disease* OR<br>≥ 3 risk factors**		+4
d. Symptoms and signs	Diaphoresis	+3
	Radiation to arm or shoulder	+5
	Pain*** occurred or worsened with inspiration	-4
	Pain* is reproduced by palpation	-6

* Coronary artery disease (CAD) = previous acute myocardial infarction, coronary artery bypass graft or percutaneous intervention.
**Risk factors = family history of premature CAD, dyslipidemia, diabetes, hypertension, current smoker.
***pain that caused presentation to hospital

Supplementary Table 3b. EDACS-ADP and recommendation

Criteria	Risk	Recommendation
- EDACS ≤16<br>- No new ischemia on ECG<br>- 0 and 2 h troponine both negative	Low	Patient safe for discharge to early outpatient follow-up investigation (or proceed to earlier inpatient testing)
- EDACS ≥ 16<br>- New ischemia on ECG<br>- Either 0 or 2 h troponin positive*	Not-low	Proceed with usual care with further observation and delayed troponin

* A 2 h troponin is only required if other parameters are low risk

c. ADAPT-ADP[6]

Supplementary Table 4a. ADAPT-ADP

**Variables**	**0 points**	**1 points**
Abnormal troponin*	No	Yes
Abnormal EKG**	No	Yes
TIMI Risk Score for UA/NSTEMI	0	>0

*cTnI level at 0 and 2 hours above institutional cutoff for elevated troponin.
**ST-segment depression of at least 0.05 mV in ≥2 contiguous leads (including reciprocal changes), T-wave inversion of at least 0.1 mV, or Q waves ≥30 ms in width and ≥0.1 mV in depth in at least 2 contiguous leads.

Supplementary Table 4b. ADAPT-ADP and recommendation

**Criteria**	**Risk group**	**Risk of MACE in 30 days**
Normal troponin, normal EKG and TIMI 0	Low	0-0.3%*
Normal troponin, normal EKG and TIMI 1	Intermediate	0.8%**

Abnormal troponin or abnormal EKG and any TIMI	High	15.3%***

*Per Than 2012.
**Per Cullen 2013. The study examined patients with TIMI 0 or 1, with no reported data for patients with TIMI >1 (presuming according to TIMI data, risk of MACE increases with increasing TIMI Score).
***Per Than 2012. In the original paper, 15.3% of patients studied (302 of 1,975 total) had a MACE, and only 1 patient was identified as low risk.

d. Troponin-only Manchester Acute Coronary Syndromes (T-MACS) Decision Aid[7]

Formula to calculate the probability of ACS
$\frac{1}{1 + e^{-(1.713E + 0.847A + 0.607R + 1.417V + 2.058S + 1.208H + 0.089T - 4.766)}}$

E = EKG ischemia, A = worsening or crescendo angina, R = Pain radiation to the right arm or shoulder, V = pain associated with vomiting, S=sweating observed, H = hypotension, T = hs-cTnT concentration on arrival

Supplementary Table 5a. T-MACS

Variables	No	Yes
E: EKG ischemia	0	1
A: Worsening or crescendo angina	0	1
R: Pain radiation to the right arm or shoulder	0	1
V: Pain associated with vomiting	0	1
S: Sweating observed	0	1
H: Hypotension	0	1
T: hs-cTnT concentration on arrival	hs-cTnT value	

Supplementary Table 5b. T-MACS and recommendation

Probability	Risk	Interpretation
<0.02	Very low	ACS ruled out. Consider discharge.
≥0.02 and <0.05	Low	Consider serial troponin in ED ward, e.g. 3h troponin; consider discharge if normal.
≥0.05 and <0.95	Moderate	Serial troponin in general ward and consider stress testing and/or CT coronary angiography thereafter.
≥0.95	High	ACS ruled in. Refer to cardiology, treat for ACS.

II. Risk scores during ACS admission

Mortality / ischemic risk

a. GRACE score[8–10]

For an overview of the GRACE score risk variables, see Figure 1. The GRACE-score webcalculators can be found online: https://www.outcomes-umassmed.org/risk_models_grace_orig.aspx

Supplementary Table 6a. GRACE risk score – in-hospital mortality.[8] Score outcomes and probabilities of in-hospital mortality.

Score	Probability in-hospital mortality
58	0.002
79	0.004
91	0.006
100	0.008
107	0.01
129	0.02
141	0.03
151	0.04
158	0.05
164	0.06
169	0.07
173	0.08
177	0.09
181	0.1
184	0.11
187	0.12
190	0.13
192	0.14
195	0.15
197	0.16
199	0.17
201	0.18
203	0.19
205	0.20
207	0.21
209	0.22
211	0.23
213	0.24
214	0.25
216	0.26
217	0.27
219	0.28
220	0.29
222	0.30
235	0.40
248	0.50
260	0.60
274	0.70
290	0.80
315	0.90

Supplementary Table 6b. GRACE risk score – admission-to-6-months mortality.[9] Score outcome and mortality risk from admission to 6 months.

Score Range	Mortality Risk
0-87	0-2%
88-128	3-10%
129-149	10-20%
150-173	20-30%
174-182	40%
183-190	50%
191-199	60%
200-207	70%
208-218	80%
219-284	90%
≥ 285	99%

b. TIMI risk score for STEMI[11]

Supplementary Table 7. TIMI risk score for STEMI and predicted mortality rate in 30 days

Variables	Outcome	Score
Age	<65 years	0
	65-74	+2
	≥75	+3

Diabetes, hypertension or angina	No	0
	Yes	+1
Systolic BP <100 mmHg	No	0
	Yes	+3
Heart rate >100	No	0
	Yes	+2
Killip Class II-IV	No	0
	Yes	+2
Weight <67 kg	No	0
	Yes	+1
Anterior ST Elevation or LBBB	No	0
	Yes	+1
Time to treatment >4 hours	No	0
	Yes	+1

**Total score**	**Mortality at 30 days (%)**
0	0.8
1	1.6
2	2.2
3	4.4
4	7.3
5	12.4
6	16.1
7	23.4
8	26.8
>8	35.9

c. TIMI risk score for UA/NSTEMI[12]

Supplementary Table 8. TIMI Risk score for UA/NSTEMI and the risk for combined end point of all-cause mortality, myocardial infarction and severe recurrent ischemia prompting urgent revascularization at 14 days.

**Variables**	**Outcome**	**Score**
Age	≥65	+1
Risk factors coronary artery disease (CAD)	≥3	+1
Known CAD (stenosis ≥ 50%)	Yes/No	+1
ASA use in past 7 days	Yes/No	+1
Severe angina	≥2 episodes in 24 hours	+1
EKG ST changes	≥0.5mm	+1
Positive cardiac marker	Yes/No	+1

**Total score**	**Combined endpoint at 14 days (%)**
0/1	4.7
2	8.3
3	13.2
4	19.9
5	26.2
6/7	40.9

d. PARIS coronary thrombotic event (CTE) score[13]

Supplementary Table 9. PARIS CTE score

Variables	Outcome	Score
Diabetes	None	0
	Type 2 diabetes	+1
	Type 1 diabetes	+3
Acute coronary syndrome	No	0
	Yes, troponin negative	+1
	Yes, troponin positive	+2
Current smoking	No	0
	Yes	+1
Creatinine clearance <60 ml/min	Absent	0
	Present	+2
Prior PCI	No	0
	Yes	+2
Prior CABG	No	0
	Yes	+2

PCI = percutaneous coronary intervention, CABG =coronary artery bypass graft.

Supplementary Table 9b. PARIS CTE score outcome and predicted thrombotic event rate

Points	Risk stratification	Predicted thrombotic event rate
0-2	Low	1.7%
3-4	Intermediate	4.10%
≥5	High	9.8%

Bleeding risk

e. **CRUSADE score**[14]

Supplementary Table 10a. Crusade score criteria, value and points.

Variables	Outcome	Score
**Baseline hematocrit**	<31	+9
	31-33.9	+7
	34-36.9	+3
	37-39.9	+2
	≥40	0
**Creatinine clearance, mL/min**	≤15	+39
	>15-30	+35
	>30-60	+28
	>60-90	+17
	>90-120	+7
	>120	0
**Heart rate**	≤70	0
	71-80	+1
	81-90	+3
	91-100	+6
	101-110	+8
	111-120	+10
	≥121	+11
**Sex**	Male	0
	Female	+8
**Signs of CHF at presentation**	No	0
	Yes	+7
**Diabetes mellitus**	No	0
	Yes	+6
**Prior vascular disease**	No	0
	Yes	+6
**Systolic blood pressure, mm Hg**	≤90	+10
	91-100	+8
	101-120	+5
	121-180	+1
	181-200	+3
	≥201	+5

Supplementary Table 10b. Crusade score and rate of major bleeding

Points	Risk stratification	Rate of major bleeding
≤20	Very low risk	3.1%
21-30	Low risk	5.5%
31-40	Moderate risk	8.6%
41-50	High risk	11.9%
>50	Very high risk	19.5%

f. **PARIS Major Bleeding (MB) score**[15]

Supplementary Table 11a. PARIS MB score

Variables	Outcome	Score
Age (years)	<50	0

	50-59	+1
	60-69	+2
	70-79	+3
	≥80	+4
BMI, kg/m^2	<25	+2
	25-34.9	0
	≥35	+2
Current smoking	No	0
	Yes	+2
Anemia	Absent	0
	Present	+3
Creatinine clearance <60 ml/min	Absent	0
	Present	+2
Triple therapy on discharge	No	0
	Yes	+2
Total score range 0 -15		

Supplementary Table 11b. PARIS CTE score and predicted bleeding rate

Points	Risk stratification	Rate of major bleeding
0-3	Low	1.4%
4-7	Intermediate	4.3%
≥8	High	9.5%

g. <u>PRECISE-DAPT score</u>[16]

For an overview of the PRECISE-DAPT variables, see Figure 1. The PRECISE-DAPT score webcalculator can be found online: http://www.precisedaptscore.com/predapt/webcalculator.html

Supplementary Table 12. PRECISE-DAPT score and recommendation

Score	Risk stratification	Recommendation
≤10	Very Low	DAPT 12-24 months
11-17	Low	DAPT 12-24 months
18-24	Moderate	DAPT 12-24 months
≥25	High	DAPT 3-6 months

h. <u>BleeMACS score</u>[17]

Supplementary Table 13a. BleeMACS score

**Variables**	**Outcome**		**Score**
Age (years)	<67		0
	67-74.9		7
	>75		9
Hypertension	Yes/No		7
Vascular disease	Yes/No		6
History of bleeding	Yes/No		19
Malignancy	Yes/No		8
Creatinine	µmol/L	g/dl	
	<88.4	<1.0	0
	88.4-131.9	1-1.49	3
	>132.0	>1.5	12
Hemoglobin	mmol/L	g/dL	
	≥6.8	<11.0	18
	6.8-8.6	11.0-13.9	9

	≥8.7	≥14	0

Table 13b. BleeMACS score outcome and risk stratification.

Points	Risk group	Bleeding events per 100 person-years
≤ 7	Very low-risk	1.48
8-16	Low-risk	2.27
17-25	Moderate-risk	3.5
≥ 26	High-risk	8.03

III. Risk scores used post-ACS

a. DAPT score[18]

Variables	Outcome	Score
Age, years	≥75	0
	65-74	-1
	<65	-2
Cigarette smoking*	Yes/No	+1
Diabetes mellitus	Yes/No	+1
MI at presentation	Yes/No	+1
Prior PCI or prior MI	Yes/No	+1
Paclitaxel-eluting stent	Yes/No	+1
Stent diameter <3 mm	Yes/No	+1
CHF or LVEF <30%	Yes/No	+2
Vein graft stent	Yes/No	+2

Supplementary Table 14a. DAPT score

*smoking within 1 year prior to index procedure.

Supplementary Table 14b. DAPT score and recommendation

DAPT Score	Net clinical benefit for extended DAPT	Recommendation
-2 to 1	No	Prolonged DAPT not recommended
≥2	Yes	Prolonged DAPT recommended

V. References used in Tables 1-3.

	Number of validation studies	Included validation studies
**HEART score**[1,2]	**16**[19]	Number of studies used in systematic review.
**EDACS**[5]	**6**[20–23]	Only studies with an end point of MACE at 30 days were included.
**T-MACS**[7]	**2**[7,24]	Only studies with an end point of MACE at 30 days were included.
**HEART Pathway**[3,4]	**7**[25–31]	Only studies with an end point of MACE at 30 days were included.
**EDACS-ADP**[5]	**6**[5,20,22,28,31,32]	Only studies with an end point of MACE at 30 days were included.
**ADAPT-ADP**[6]	**5**[26,28,33–35]	Only studies with an end point of MACE at 30 days were included.

**GRACE – in-hospital**[8]	**17**[8,36–51]	Only studies with an end point of in-hospital mortality were included.
**GRACE – 6 months**[9]	**6**[9,39,46,52–54]	Only studies with an end point of mortality at 6 months were included.
**TIMI (UA/NSTEMI)**[11]	**19**[12,55–72]	Only studies with a combined end point of death, MI or revascularization at 14 days were included.
**TIMI (STEMI)**[12]	**16**[73–86]	Only studies with an end point of mortality at 30 days were included.
**PARIS CTE**[15]	**6**[15,87–91]	Only studies that included MI or ST in the combined end point were included.

**CRUSADE**[14]	**8**[14,37,92–97]	Only studies with an end point of in-hospital bleeding were included.
**PARIS MB**[15]	**7**[15,87,89,90,98–100]	Only studies with an endpoint of moderate or severe bleeding were included.
**PRECISE-DAPT**[16]	**6**[16,90,98–101]	Only studies with an end point of moderate or severe bleeding were included.
**BleeMACS**[17]	**2**[17,99]	Only studies with an end point of moderate or severe bleeding were included.

**DAPT score –ischemic model**[18]	**2**[18,102]	Only studies that analyzed the separate ischemic model of the DAPT-score with an endpoint of MI or ST at 30 months were included.
**DAPT score –bleeding model**[18]	**2**[18,102]	Only studies that analyzed the separate bleeding model of the DAPT-score with an endpoint of moderate or severe bleeding at 30 months were included.
**DAPT score – Final risk score – ischemic outcome**[18]	**6**[87,102–106]	Studies that analyzed a long-term ischemic outcome were included.
**DAPT score – Final risk score – bleeding outcome**[18]	**6**[87,102–106]	Studies that analyzed a long-term bleeding outcome were included.

Reference list

1. Six AJ, Backus BE, Kelder JC. Heart Score Value. *Netherlands Hear J*. 2008;16(6):191-196.
2. Backus BE, Six AJ, Kelder JC, et al. Chest pain in the emergency room: a multicenter validation of the HEART Score. *Crit Pathw Cardiol*. 2010;9(3):164-169. doi:10.1097/HPC.0b013e3181ec36d8
3. Mahler SA, Hiestand BC, Goff DCJ, Hoekstra JW, Miller CD. Can the HEART score safely reduce stress testing and cardiac imaging in patients at low risk for major adverse cardiac events? *Crit Pathw Cardiol*. 2011;10(3):128-133. doi:10.1097/HPC.0b013e3182315a85
4. Mahler SA, Miller CD, Hollander JE, et al. Identifying patients for early discharge: Performance of decision rules among patients with acute chest pain. *Int J Cardiol*. 2013;168(2):795-802. doi:10.1016/j.ijcard.2012.10.010
5. Than M, Flaws D, Sanders S, et al. Development and validation of the Emergency Department Assessment of Chest pain Score and 2 h accelerated diagnostic protocol. *Emerg Med Australas*. 2014;26(1):34-44. doi:10.1111/1742-6723.12164
6. Than M, Cullen L, Aldous S, et al. 2-Hour accelerated diagnostic protocol to assess patients with chest pain symptoms using contemporary troponins as the only biomarker: the ADAPT trial. *J Am Coll Cardiol*. 2012;59(23):2091-2098. doi:10.1016/j.jacc.2012.02.035
7. Body R, Carlton E, Sperrin M, et al. Troponin-only Manchester Acute Coronary Syndromes (T-MACS) decision aid: single biomarker re-derivation and external validation in three cohorts. *Emerg Med J*. 2017;34(6):349-356. doi:10.1136/emermed-2016-205983
8. Granger CB, Goldberg RJ, Dabbous O, et al. Predictors of hospital mortality in the global registry of acute coronary events. *Arch Intern Med*. 2003;163(19):2345-2353. doi:10.1001/archinte.163.19.2345
9. Fox KAA, Dabbous OH, Goldberg RJ, et al. Prediction of risk of death and myocardial infarction in the six months after presentation with acute coronary syndrome: Prospective multinational observational study (GRACE). *Br Med J*. 2006;333(7578):1091-1094. doi:10.1136/bmj.38985.646481.55
10. Fox KAA, FitzGerald G, Puymirat E, et al. Should patients with acute coronary disease be stratified for management according to their risk? Derivation, external validation and outcomes using the updated GRACE risk score. *BMJ Open*. 2014;4(2):e004425-2013-004425. doi:10.1136/bmjopen-2013-004425
11. Morrow DA, Antman EM, Charlesworth A, et al. TIMI risk score for ST-elevation myocardial infarction: A convenient, bedside, clinical score for risk assessment at presentation: An Intravenous nPA for Treatment of Infarcting Myocardium Early II trial substudy. *Circulation*. 2000;102(17):2031-2037. doi:10.1161/01.CIR.102.17.2031
12. Antman EM, Cohen M, Bernink PJLM, et al. The TIMI risk score for unstable angina/non-ST elevation MI: A method for prognostication and therapeutic decision making. *J Am Med Assoc*. 2000;284(7):835-842. doi:10.1001/jama.284.7.835
13. Zhao X, Li J, Tang X, et al. Prognostic Value of the PARIS Thrombotic Risk Score for 2-Year Mortality After Percutaneous Coronary Intervention. *Clin Appl Thromb*. 2019;25(167). doi:10.1177/1076029619853638
14. Subherwal S, Bach RG, Chen AY, et al. Baseline risk of major bleeding in non-sT-segment- elevation myocardial infarction the CRUSADE (can rapid risk stratification of unstable angina patients suppress ADverse outcomes with early implementation of the ACC/AHA guidelines) bleeding score. *Circulation*. 2009;119(14):1873-1882. doi:10.1161/CIRCULATIONAHA.108.828541
15. Baber U, Mehran R, Giustino G, et al. Coronary Thrombosis and Major Bleeding after PCI with Drug-Eluting Stents Risk Scores from Paris. *J Am Coll Cardiol*. 2016;67(19):2224-2234. doi:10.1016/j.jacc.2016.02.064
16. Costa F, van Klaveren D, James S, et al. Derivation and validation of the predicting bleeding complications in patients undergoing stent implantation and subsequent dual antiplatelet therapy (PRECISE-DAPT) score: a pooled analysis of individual-patient datasets from clinical trials. *Lancet*. 2017;389(10073):1025-1034. doi:10.1016/S0140-6736(17)30397-5
17. Raposeiras-Roubín S, Faxén J, Íñiguez-Romo A, et al. Development and external validation of a post-discharge bleeding risk score in patients with acute coronary syndrome: The BleeMACS score. *Int J Cardiol*. 2018;254:10-15. doi:10.1016/j.ijcard.2017.10.103
18. Yeh RW, Secemsky EA, Kereiakes DJ, et al. Development and validation of a prediction rule for benefit and harm of Dual antiplatelet therapy beyond 1 year after percutaneous coronary intervention. *JAMA - J Am Med Assoc*. 2016;315(16):1735-1749. doi:10.1001/jama.2016.3775
19. Laureano-Phillips J, Robinson RD, Aryal S, et al. HEART Score Risk Stratification of Low-Risk Chest Pain Patients in the Emergency Department: A Systematic Review and Meta-Analysis. *Ann Emerg Med*. 2019;74(2):187-203. doi:10.1016/j.annemergmed.2018.12.010
20. Flaws D, Than M, Scheuermeyer FX, et al. External validation of the emergency department assessment of

chest pain score accelerated diagnostic pathway (EDACS-ADP). *Emerg Med J*. 2016;33(9):618-625. doi:10.1136/emermed-2015-205028

21. Sanders S, Flaws D, Than M, Pickering JW, Doust J, Glasziou P. Simplification of a scoring system maintained overall accuracy but decreased the proportion classified as low risk. *J Clin Epidemiol*. 2016;69(2016):32-39. doi:10.1016/j.jclinepi.2015.05.006
22. Shin YS, Ahn S, Kim Y-J, Ryoo SM, Sohn CH, Kim WY. External validation of the emergency department assessment of chest pain score accelerated diagnostic pathway (EDACS-ADP). *Am J Emerg Med*. 2019;(xxxx). doi:10.1016/j.ajem.2019.09.019
23. Shin YS, Ahn S, Kim YJ, Ryoo SM, Sohn CH, Kim WY. Risk stratification of patients with chest pain or anginal equivalents in the emergency department. *Intern Emerg Med*. 2020;15(2):319-326. doi:10.1007/s11739-019-02230-0
24. Va Den Berg P, Burrows G, Lewis P, Carley S, Body R. Validation of the (Troponin-only) Manchester ACS decision aid with a contemporary cardiac troponin I assay. *Am J Emerg Med*. 2018;36(4):602-607. doi:10.1016/j.ajem.2017.09.032
25. Mahler SA, Riley RF, Hiestand BC, et al. The HEART Pathway Randomized Trial: Identifying Emergency Department Patients with Acute Chest Pain for Early Discharge. *Circ Cardiovasc Qual Outcomes*. 2015;8(2):195-203. doi:10.1161/CIRCOUTCOMES.114.001384
26. Stopyra JP, Miller CD, Hiestand BC, et al. Chest pain risk stratification a comparison of the 2-hour accelerated diagnostic protocol (ADAPT) and the HEART pathway. *Crit Pathw Cardiol*. 2016;15(2):46-49. doi:10.1097/HPc.0000000000000072
27. Mahler SA, Lenoir KM, Wells BJ, et al. Safely Identifying Emergency Department Patients with Acute Chest Pain for Early Discharge: HEART Pathway Accelerated Diagnostic Protocol. *Circulation*. 2018;138(22):2456-2468. doi:10.1161/CIRCULATIONAHA.118.036528
28. Greenslade JH, Carlton EW, Van Hise C, et al. Diagnostic Accuracy of a New High-Sensitivity Troponin I Assay and Five Accelerated Diagnostic Pathways for Ruling Out Acute Myocardial Infarction and Acute Coronary Syndrome. *Ann Emerg Med*. 2018;71(4):439-451.e3. doi:10.1016/j.annemergmed.2017.10.030
29. Oliver JJ, Streitz MJ, Hyams JM, et al. An external validation of the HEART pathway among Emergency Department patients with chest pain. *Intern Emerg Med*. 2018;13(8):1249-1255. doi:10.1007/s11739-018-1809-y
30. Tesson A, Abdo R, Kamath A, et al. Heart failure? Episodes of missed major cardiac events when applying the heart pathway to an observation unit population. *Crit Pathw Cardiol*. 2018;17(2):88-94. doi:10.1097/HPC.0000000000000137
31. Stopyra J, Snavely AC, Hiestand B, et al. Comparison of accelerated diagnostic pathways for acute chest pain risk stratification. *Heart*. 2020;106(13):977-984. doi:10.1136/heartjnl-2019-316426
32. Stopyra JP, Miller CD, Hiestand BC, et al. Performance of the EDACS-accelerated diagnostic pathway in a cohort of US patients with acute chest pain. *Crit Pathw Cardiol*. 2015;14(4):134-138. doi:10.1097/HPC.0000000000000059
33. Cullen L, Mueller C, Parsonage WA, et al. Validation of high-sensitivity troponin I in a 2-hour diagnostic strategy to assess 30-day outcomes in emergency department patients with possible acute coronary syndrome. *J Am Coll Cardiol*. 2013;62(14):1242-1249. doi:10.1016/j.jacc.2013.02.078
34. Fanaroff AC, Schulteis RD, Pieper KS, Rao S V, Newby LK. Simplified Predictive Instrument to Rule Out Acute Coronary Syndromes in a High-Risk Population. *J Am Heart Assoc*. 2015;4(12). doi:10.1161/JAHA.115.002351
35. Sepehrvand N, Zheng Y, Armstrong PW, Welsh RC, Ezekowitz JA. Identifying Low-risk Patients for Early Discharge From Emergency Department Without Using Subjective Descriptions of Chest Pain: Insights From Providing Rapid Out of Hospital Acute Cardiovascular Treatment (PROACT) 3 and 4 Trials. *Acad Emerg Med Off J Soc Acad Emerg Med*. 2017;24(6):691-700. doi:10.1111/acem.13183
36. Yan AT, Yan RT, Tan M, et al. Risk scores for risk stratification in acute coronary syndromes: useful but simpler is not necessarily better. *Eur Heart J*. 2007;28(9):1072-1078. doi:10.1093/eurheartj/ehm004
37. Manzano-Fernández S, Sánchez-Martínez M, Flores-Blanco PJ, et al. Comparison of the Global Registry of Acute Coronary Events Risk Score Versus the Can Rapid Risk Stratification of Unstable Angina Patients Suppress Adverse outcomes With Early Implementation of the ACC/AHA Guidelines Risk Score to Predict In-Hospital Mor. *Am J Cardiol*. 2016;117(7):1047-1054. doi:10.1016/j.amjcard.2015.12.048
38. Baeza-Román A, de Miguel-Balsa E, Latour-Pérez J, Carrillo-López A. Predictive power of the grace score in population with diabetes. *Int J Cardiol*. 2017;248(2017):73-76. doi:10.1016/j.ijcard.2017.06.083
39. Chen Y-H, Huang S-S, Lin S-J. TIMI and GRACE Risk Scores Predict Both Short-Term and Long-Term Outcomes in Chinese Patients with Acute Myocardial Infarction. *Acta Cardiol Sin*. 2018;34(1):4-12.

doi:10.6515/ACS.201801_34(1).20170730B
40. Cerqueira Junior AM dos S, Pereira LG dos S, De Souza TMB, et al. Prognostic accuracy of the grace score in octogenarians and nonagenarians with acute coronary syndromes. *Arq Bras Cardiol*. 2018;110(1):24-29. doi:10.5935/abc.20170175
41. Komiyama K, Nakamura M, Tanabe K, et al. In-hospital mortality analysis of Japanese patients with acute coronary syndrome using the Tokyo CCU Network database: Applicability of the GRACE risk score. *J Cardiol*. 2018;71(3):251-258. doi:10.1016/j.jjcc.2017.09.006
42. Kwon J-M, Jeon K-H, Kim HM, et al. Deep-learning-based risk stratification for mortality of patients with acute myocardial infarction. *PLoS One*. 2019;14(10):e0224502. doi:10.1371/journal.pone.0224502
43. Tscherny K, Kienbacher C, Fuhrmann V, et al. Risk stratification in acute coronary syndrome: Evaluation of the GRACE and CRUSADE scores in the setting of a tertiary care centre. *Int J Clin Pract*. 2020;74(2):e13444. doi:10.1111/ijcp.13444
44. Elbarouni B, Goodman SG, Yan RT, et al. Validation of the Global Registry of Acute Coronary Event (GRACE) risk score for in-hospital mortality in patients with acute coronary syndrome in Canada. *Am Heart J*. 2009;158(3):392-399. doi:10.1016/j.ahj.2009.06.010
45. Aragam KG, Tamhane UU, Kline-Rogers E, et al. Does simplicity compromise accuracy in ACS risk prediction? A retrospective analysis of the TIMI and GRACE risk scores. *PLoS One*. 2009;4(11):1-9. doi:10.1371/journal.pone.0007947
46. Abu-Assi E, Ferreira-González I, Ribera A, et al. Do GRACE (Global Registry of Acute Coronary events) risk scores still maintain their performance for predicting mortality in the era of contemporary management of acute coronary syndromes? *Am Heart J*. 2010;160(5). doi:10.1016/j.ahj.2010.06.053
47. Chan MY, Shah BR, Gao F, et al. Recalibration of the Global Registry of Acute Coronary Events risk score in a multiethnic Asian population. *Am Heart J*. 2011;162(2):291-299. doi:10.1016/j.ahj.2011.05.016
48. Meune C, Drexler B, Haaf P, et al. The GRACE score's performance in predicting in-hospital and 1-year outcome in the era of high-sensitivity cardiac troponin assays and B-type natriuretic peptide. *Heart*. 2011;97(18):1479-1483. doi:10.1136/hrt.2010.220988
49. Scruth EA, Cheng E, Worrall-Carter L. Risk score comparison of outcomes in patients presenting with ST-elevation myocardial infarction treated with percutaneous coronary intervention. *Eur J Cardiovasc Nurs*. 2013;12(4):330-336. doi:10.1177/1474515112449412
50. Shaikh MK, Hanif B, Shaikh K, Khan W, Parkash J. Validation of grace risk score in predicting in-hospital mortality in patients with non ST-elevation myocardial infarction and unstable angina. *J Pak Med Assoc*. 2014;64(7):807-811.
51. Fujii T, Suzuki T, Torii S, et al. Diagnostic accuracy of global registry of acute coronary events (GRACE) risk score in ST-Elevation myocardial infarction for in-hospital and 360-day mortality in Japanese patients. *Circ J*. 2014;78(12):2950-2954. doi:10.1253/circj.CJ-14-0808
52. Tang EW, Wong CK, Herbison P. Global Registry of Acute Coronary Events (GRACE) hospital discharge risk score accurately predicts long-term mortality post acute coronary syndrome. *Am Heart J*. 2007;153(1):29-35. doi:10.1016/j.ahj.2006.10.004
53. Littnerova S, Kala P, Jarkovsky J, et al. GRACE Score among Six Risk Scoring Systems (CADILLAC, PAMI, TIMI, Dynamic TIMI, Zwolle) Demonstrated the Best Predictive Value for Prediction of Long-Term Mortality in Patients with ST-Elevation Myocardial Infarction. *PLoS One*. 2015;10(4):e0123215. doi:10.1371/journal.pone.0123215
54. Kao YT, Hsieh YC, Hsu CY, et al. Comparison of the TIMI, GRACE, PAMI and CADILLAC risk scores for prediction of longterm cardiovascular outcomes in Taiwanese diabetic patients with ST-segment elevation myocardial infarction: From the registry of the Taiwan Society of Cardiology. *PLoS One*. 2020;15(2):1-13. doi:10.1371/journal.pone.0229186
55. Morrow DA, Antman EM, Snapinn SM, McCabe CH, Theroux P, Braunwald E. An integrated clinical approach to predicting the benefit of tirofiban in non-ST elevation acute coronary syndromes. Application of the TIMI Risk Score for UA/NSTEMI in PRISM-PLUS. *Eur Heart J*. 2002;23(3):223-229. doi:10.1053/euhj.2001.2738
56. Stähli BE, Yonekawa K, Altwegg LA, et al. Clinical criteria replenish high-sensitive troponin and inflammatory markers in the stratification of patients with suspected acute coronary syndrome. *PLoS One*. 2014;9(6). doi:10.1371/journal.pone.0098626
57. Macdonald SPJ, Nagree Y, Fatovich DM, Brown SGA. Modified TIMI risk score cannot be used to identify low-risk chest pain in the emergency department: A multicentre validation study. *Emerg Med J*. 2014;31(4):281-285. doi:10.1136/emermed-2012-201323
58. Liu N, Lee MAB, Ho AFW, et al. Risk stratification for prediction of adverse coronary events in

emergency department chest pain patients with a machine learning score compared with the TIMI score. *Int J Cardiol*. 2014;177(3):1095-1097. doi:10.1016/j.ijcard.2014.09.199
59. Boubaker H, Grissa MH, Beltaief K, et al. A new score for the diagnosis of acute coronary syndrome in acute chest pain with non-diagnostic ECG and normal troponin. *Emerg Med J*. 2015;32(10):764-768. doi:10.1136/emermed-2013-203151
60. Jain T, Nowak R, Hudson M, Frisoli T, Jacobsen G, McCord J. Short- and Long-Term Prognostic Utility of the HEART Score in Patients Evaluated in the Emergency Department for Possible Acute Coronary Syndrome. *Crit Pathw Cardiol*. 2016;15(2):40-45. doi:10.1097/HPC.0000000000000070
61. Heldeweg MLA, Liu N, Koh ZX, et al. A novel cardiovascular risk stratification model incorporating ECG and heart rate variability for patients presenting to the emergency department with chest pain. *Crit Care*. 2016;20(1):179. doi:10.1186/s13054-016-1367-5
62. Rainer TH, Leung YK, Lee A, et al. Add-on tests for improving risk-stratification in emergency department patients with chest pain who are at low to moderate risk of 30-day major adverse cardiac events. *Int J Cardiol*. 2016;220(2016):299-306. doi:10.1016/j.ijcard.2016.05.057
63. Wong CP, Lui CT, Sung JG, Lam H, Fung HT, Yam PW. Prognosticating Clinical Prediction Scores Without Clinical Gestalt for Patients With Chest Pain in the Emergency Department. *J Emerg Med*. 2018;54(2):176-185. doi:10.1016/j.jemermed.2017.10.006
64. Roongsritong C, Taha ME, Pisipati S, et al. SVEAT Score, a Potential New and Improved Tool for Acute Chest Pain Risk Stratification. *Am J Cardiol*. April 2020. doi:10.1016/j.amjcard.2020.04.009
65. Samaha FF, Kimmel SE, Kizer JR, Goyal A, Wade M, Boden WE. Usefulness of the TIMI risk score in predicting both short- and long-term outcomes in the Veterans Affairs Non-Q-Wave Myocardial Infarction Strategies In-Hospital (VANQWISH) Trial. *Am J Cardiol*. 2002;90(9):922-926. doi:10.1016/S0002-9149(02)02654-1
66. Sanchis J, Bodí V, Núñez J, et al. New risk score for patients with acute chest pain, non-ST-segment deviation, and normal troponin concentrations: A comparison with the TIMI risk score. *J Am Coll Cardiol*. 2005;46(3):443-449. doi:10.1016/j.jacc.2005.04.037
67. Jaffery Z, Hudson MP, Jacobsen G, Nowak R, McCord J. Modified thrombolysis in myocardial infarction (TIMI) risk score to risk stratify patients in the emergency department with possible acute coronary syndrome. *J Thromb Thrombolysis*. 2007;24(2):137-144. doi:10.1007/s11239-007-0013-0
68. Body R, Carley S, McDowell G, Ferguson J, Mackway-Jones K. Can a modified thrombolysis in myocardial infarction risk score outperform the original for risk stratifying emergency department patients with chest pain? *Emerg Med J*. 2009;26(2):95-99. doi:10.1136/emj.2008.058495
69. Hess EP, Perry JJ, Calder LA, et al. Prospective validation of a modified thrombolysis in myocardial infarction risk score in emergency department patients with chest pain and possible acute coronary syndrome. *Acad Emerg Med*. 2010;17(4):368-375. doi:10.1111/j.1553-2712.2010.00696.x
70. Lee B, Chang AM, Matsuura AC, Marcoon S, Hollander JE. Comparison of cardiac risk scores in ED patients with potential acute coronary syndrome. *Crit Pathw Cardiol*. 2011;10(2):64-68. doi:10.1097/HPC.0b013e31821c79bd
71. Macdonald SPJ, Nagree Y, Fatovich DM, Flavell HL, Loutsky F. Comparison of two clinical scoring systems for emergency department risk stratification of suspected acute coronary syndrome. *Emerg Med Australas*. 2011;23(6):717-725. doi:10.1111/j.1742-6723.2011.01480.x
72. Six AJ, Cullen L, Backus BE, et al. The HEART score for the assessment of patients with chest pain in the emergency department: a multinational validation study. *Crit Pathw Cardiol*. 2013;12(3):121-126. doi:10.1097/HPC.0b013e31828b327e
73. Lev EI, Kornowski R, Vaknin-Assa H, et al. Comparison of the Predictive Value of Four Different Risk Scores for Outcomes of Patients With ST-Elevation Acute Myocardial Infarction Undergoing Primary Percutaneous Coronary Intervention. *Am J Cardiol*. 2008;102(1):6-11. doi:10.1016/j.amjcard.2008.02.088
74. Kozieradzka A, Kamiński KA, Maciorkowska D, et al. GRACE, TIMI, Zwolle and CADILLAC risk scores - Do they predict 5-year outcomes after ST-elevation myocardial infarction treated invasively? *Int J Cardiol*. 2011;148(1):70-75. doi:10.1016/j.ijcard.2009.10.026
75. Damman P, Woudstra P, Kuijt WJ, et al. Short- and long-term prognostic value of the TIMI risk score after primary percutaneous coronary intervention for ST-segment elevation myocardial infarction. *J Interv Cardiol*. 2013;26(1):8-13. doi:10.1111/j.1540-8183.2012.00763.x
76. Tesak M, Kala P, Jarkovsky J, et al. The value of novel invasive hemodynamic parameters added to the TIMI risk score for short-term prognosis assessment in patients with ST segment elevation myocardial infarction. *Int J Cardiol*. 2016;214(2016):235-240. doi:10.1016/j.ijcard.2016.03.073
77. Popovic B, Girerd N, Rossignol P, et al. Prognostic Value of the Thrombolysis in Myocardial Infarction

Risk Score in ST-Elevation Myocardial Infarction Patients With Left Ventricular Dysfunction (from the EPHESUS Trial). *Am J Cardiol*. 2016;118(10):1442-1447. doi:10.1016/j.amjcard.2016.08.004
78. Shouval R, Hadanny A, Shlomo N, et al. Machine learning for prediction of 30-day mortality after ST elevation myocardial infraction: An Acute Coronary Syndrome Israeli Survey data mining study. *Int J Cardiol*. 2017;246(2017):7-13. doi:10.1016/j.ijcard.2017.05.067
79. Morrow DA, Antman EM, Charlesworth A, et al. TIMI risk score for ST-elevation myocardial infarction: A convenient, bedside, clinical score for risk assessment at presentation: An intravenous nPA for treatment of infarcting myocardium early II trial substudy. *Circulation*. 2000;102(17):2031-2037. doi:10.1161/01.cir.102.17.2031
80. Singh M, Reeder GS, Jacobsen SJ, Weston S, Killian J, Roger VL. Scores for post-myocardial infarction risk stratification in the community. *Circulation*. 2002;106(18):2309-2314. doi:10.1161/01.cir.0000036598.12888.de
81. Morrow DA, Antman EM, Murphy SA, et al. The Risk Score Profile: a novel approach to characterising the risk of populations enrolled in clinical studies. *Eur Heart J*. 2004;25(13):1139-1145. doi:10.1016/j.ehj.2004.04.036
82. Rathore SS, Weinfurt KP, Foody JAM, Krumholz HM. Performance of the Thrombolysis in Myocardial Infarction (TIMI) ST-elevation myocardial infarction risk score in a national cohort of elderly patients. *Am Heart J*. 2005;150(3):402-410. doi:10.1016/j.ahj.2005.03.069
83. Khan SQ, Quinn P, Davies JE, Ng LL. N-terminal pro-B-type natriuretic peptide is better than TIMI risk score at predicting death after acute myocardial infarction. *Heart*. 2008;94(1):40-43. doi:10.1136/hrt.2006.108985
84. Dominguez-Rodriguez A, Abreu-Gonzalez P, Jimenez-Sosa A, Samimi-Fard S, Idaira H-B. Does ischemia-modified albumin add prognostic value to the Thrombolysis In Myocardial Infarction risk score in patients with ST-segment elevation myocardial infarction treated with primary angioplasty? *Biomarkers Biochem Indic Expo response, susceptibility to Chem*. 2009;14(1):43-48. doi:10.1080/13547500802706020
85. Méndez-Eirín E, Flores-Ríos X, García-López F, et al. Comparison of the prognostic predictive value of the TIMI, PAMI, CADILLAC, and GRACE risk scores in STEACS undergoing primary or rescue PCI. *Rev Esp Cardiol (Engl Ed)*. 2012;65(3):227-233. doi:10.1016/j.recesp.2011.10.019
86. Yang JH, Song PS, Song Y Bin, et al. Prognostic value of admission blood glucose level in patients with and without diabetes mellitus who sustain ST segment elevation myocardial infarction complicated by cardiogenic shock. *Crit Care*. 2013;17(5):R218. doi:10.1186/cc13035
87. Song L, Guan C, Yan H, et al. Validation of contemporary risk scores in predicting coronary thrombotic events and major bleeding in patients with acute coronary syndrome after drug-eluting stent implantations. *Catheter Cardiovasc Interv*. 2018;91(S1):573-581. doi:10.1002/ccd.27468
88. Zhao XY, Li JX, Tang XF, et al. Evaluation of the Patterns of Non-Adherence to Anti-Platelet Regimens in Stented Patients Bleeding Score for Predicting the Long-term Out-of-hospital Bleeding Risk in Chinese Patients after Percutaneous Coronary Intervention. *Chin Med J (Engl)*. 2018;131(12):1406-1411. doi:10.4103/0366-6999.228766
89. Raposeiras-Roubín S, Caneiro Queija B, D'Ascenzo F, et al. Usefulness of the PARIS Score to Evaluate the Ischemic-hemorrhagic Net Benefit With Ticagrelor and Prasugrel After an Acute Coronary Syndrome. *Rev Esp Cardiol*. 2019;72(3):215-223. doi:10.1016/j.recesp.2018.02.008
90. Bianco M, D'ascenzo F, Raposeiras Roubin S, et al. Comparative external validation of the PRECISE-DAPT and PARIS risk scores in 4424 acute coronary syndrome patients treated with prasugrel or ticagrelor. *Int J Cardiol*. 2020;301:200-206. doi:10.1016/j.ijcard.2019.11.132
91. Gallone G, D'Ascenzo F, Conrotto F, et al. Accuracy of the PARIS score and PCI complexity to predict ischemic events in patients treated with very thin stents in unprotected left main or coronary bifurcations. *Catheter Cardiovasc Interv Off J Soc Card Angiogr Interv*. May 2020. doi:10.1002/ccd.28972
92. Abu-Assi E, Gracía-Acuña JM, Ferreira-González I, Peña-Gil C, Gayoso-Diz P, González-Juanatey JR. Evaluating the Performance of the Can Rapid Risk Stratification of Unstable Angina Patients Suppress Adverse Outcomes With Early Implementation of the ACC/AHA Guidelines (CRUSADE) bleeding score in a contemporary Spanish cohort of patients with non-ST-se. *Circulation*. 2010;121(22):2419-2426. doi:10.1161/CIRCULATIONAHA.109.925594
93. Abu-Assi E, Raposeiras-Roubin S, Lear P, et al. Comparing the predictive validity of three contemporary bleeding risk scores in acute coronary syndrome. *Eur Hear J Acute Cardiovasc Care*. 2012;1(3):222-231. doi:10.1177/2048872612453924
94. Ariza-Solé A, Sánchez-Elvira G, Sánchez-Salado JC, et al. CRUSADE bleeding risk score validation for ST-segment-elevation myocardial infarction undergoing primary percutaneous coronary intervention. *Thromb Res*. 2013;132(6):652-658. doi:10.1016/j.thromres.2013.09.019

95. Ariza-Solé A, Salazar-Mendiguchía J, Lorente V, et al. Predictive ability of bleeding risk scores in the routine clinical practice. *Eur Hear journal Acute Cardiovasc care*. 2015;4(3):205-210. doi:10.1177/2048872614538405
96. Flores-Ríos X, Couto-Mallón D, Rodríguez-Garrido J, et al. Comparison of the performance of the CRUSADE, ACUITY-HORIZONS, and ACTION bleeding risk scores in STEMI undergoing primary PCI: Insights from a cohort of 1391 patients. *Eur Hear J Acute Cardiovasc Care*. 2013;2(1):19-26. doi:10.1177/2048872612469885
97. Liu R, Zheng W, Zhao G, et al. Predictive validity of CRUSADE, ACTION and ACUITY-HORIZONS bleeding risk scores in chinese patients with ST-segment elevation myocardial infarction. *Circ J*. 2018;82(3):791-797. doi:10.1253/circj.CJ-17-0760
98. Abu-Assi E, Raposeiras-Roubin S, Cobas-Paz R, et al. Assessing the performance of the PRECISE-DAPT and Paris risk scores for predicting one-year out-of-hospital bleeding in acute coronary syndrome patients. *EuroIntervention*. 2018;13(16):1914-1922. doi:10.4244/EIJ-D-17-00550
99. Pavasini R, Maietti E, Tonet E, et al. Bleeding Risk Scores and Scales of Frailty for the Prediction of Haemorrhagic Events in Older Adults with Acute Coronary Syndrome: Insights from the FRASER study. *Cardiovasc drugs Ther*. 2019;33(5):523-532. doi:10.1007/s10557-019-06911-y
100. Ueki Y, Bar S, Losdat S, et al. Validation of Bleeding Risk Criteria (ARC-HBR) in Patients Undergoing Percutaneous Coronary Intervention and Comparison with Contemporary Bleeding Risk Scores. *EuroIntervention J Eur Collab with Work Gr Interv Cardiol Eur Soc Cardiol*. February 2020. doi:10.4244/EIJ-D-20-00052
101. Choi SY, Kim MH, Cho YR, et al. Performance of PRECISE-DAPT Score for Predicting Bleeding Complication During Dual Antiplatelet Therapy. *Circ Cardiovasc Interv*. 2018;11(12):e006837. doi:10.1161/CIRCINTERVENTIONS.118.006837
102. Ueda P, Jernberg T, James S, et al. External Validation of the DAPT Score in a Nationwide Population. *J Am Coll Cardiol*. 2018;72(10):1069-1078. doi:10.1016/j.jacc.2018.06.023
103. Harada Y, Michel J, Lohaus R, et al. Validation of the DAPT score in patients randomized to 6 or 12 months clopidogrel after predominantly second-generation drug-eluting stents. *Thromb Haemost*. 2017;117(10):1989-1999. doi:10.1160/TH17-02-0101
104. Veron-Esquivel D, Batiz-Armenta F, Cazares-Diazleal AC, et al. Validation of DAPT score for prolonged dual antiplatelet therapy in patients with acute myocardial infarction. *Hell J Cardiol*. 2018. doi:10.1016/j.hjc.2018.05.007
105. Brener SJ, Kirtane AJ, Rinaldi MJ, et al. Prediction of ischemic and bleeding events using the dual antiplatelet therapy score in an unrestricted percutaneous coronary intervention population: Analysis from the ADAPT-DES registry. *Circ Cardiovasc Interv*. 2018;11(10):1-9. doi:10.1161/CIRCINTERVENTIONS.118.006853
106. Witberg G, Zusman O, Bental T, et al. Validation of the DAPT score in real-world patients undergoing coronary stent implantation. *Int J Cardiol*. 2019;(xxxx). doi:10.1016/j.ijcard.2019.08.044
